# Exploring Key Regulators of Mitochondrial Dynamics and Immune Response in SARS-CoV-2 Infection

**DOI:** 10.3390/v18060675

**Published:** 2026-06-16

**Authors:** Thatiana Corrêa de Melo, Hellen Paula Valerio, Dilza Trevisan-Silva, Marcelo Medina de Souza, Amanda Teixeira de Melo, Miryam Paola Alvarez-Flores, Douglas Souza Oliveira, Renata Nascimento Gomes, Glaucia Maria Machado-Santelli, Beatriz Fumelli Monti Ribeiro, Viviane Fongaro Botosso, Soraia Attie Calil Jorge, Ana Marisa Chudzinski-Tavassi

**Affiliations:** 1 Centre of Excellence in New Target Discovery (CENTD), Butantan Institute, São Paulo 05503-900, Brazil; hellenpvalerio@gmail.com (H.P.V.); dilzatrevisan@gmail.com (D.T.-S.); marcelo.msouza@alumni.usp.br (M.M.d.S.); a.melo.proppg@proppg.butantan.gov.br (A.T.d.M.); miryam.flores@butantan.gov.br (M.P.A.-F.); douglas.oliveira@butantan.gov.br (D.S.O.); renata.gomes@fundacaobutantan.org.br (R.N.G.); 2 Department of Cell and Developmental Biology, Institute of Biomedical Sciences (ICB-USP), University of São Paulo, São Paulo 05508-000, Brazil; glaucia.usp@gmail.com (G.M.M.-S.); beatrizfumelli@gmail.com (B.F.M.R.); 3 Virology Laboratory, Butantan Institute, São Paulo 05503-900, Brazil; viviane.botosso@butantan.gov.br; 4 Viral Biotechnology Laboratory, Butantan Institute, São Paulo 05503-900, Brazil; soraia.jorge@butantan.gov.br

**Keywords:** SARS-CoV-2, ISG15, SUMO2/3, MFN2, mitochondrial dynamics, MAVS, post-translational modifications, proteomics, innate immunity, LC-HK2 cells

## Abstract

Mitochondria are central hubs of antiviral immunity and cellular metabolism, yet the links between SARS-CoV-2–induced mitochondrial remodeling, antiviral gene regulation, and post-translational control remain incompletely understood. Here, we investigated mitochondrial–immune remodeling in SARS-CoV-2–infected lung-derived LC-HK2 cells at 48 and 96 h post-infection using confocal and high-content imaging, colocalization analysis, CellProfiler quantification, RT-qPCR, proteomics, cytokine profiling, and conditioned-medium analysis. Infection induced a time-dependent mitochondrial phenotype. At 48 hpi, cells displayed early mitochondrial stress and fission-associated signatures, including increased DRP1, transient upregulation of mitochondrial respiratory genes, and reduced MFN1/2. At 96 hpi, mitochondria shifted toward elongated perinuclear networks, accompanied by increased fusion/biogenesis markers and partial ISG15–MFN2 colocalization, indicating a spatial association between ISG15-related antiviral/stress responses and mitochondrial remodeling. Antiviral and ISG-related transcripts were consistently upregulated, but IFN-α2 secretion remained limited, suggesting partial uncoupling between antiviral transcriptional activation and downstream interferon output. SUMO2/3 was dynamically modulated and showed time-dependent colocalization with mitochondrial dynamics proteins and MAVS. Together, these data support a coordinated mitochondrial–immune regulatory axis involving mitochondrial remodeling, ISG15-associated responses, and SUMO-dependent regulation during SARS-CoV-2 infection.

## 1. Introduction

Coronavirus disease 2019 (COVID-19), caused by severe acute respiratory syndrome coronavirus 2 (SARS-CoV-2), has triggered an unprecedented global health crisis with profound respiratory and systemic consequences. While a large proportion of infections are mild or asymptomatic, individuals with underlying comorbidities face a substantially increased risk of developing severe pulmonary and multi-organ complications [1]. Despite the vast amount of knowledge gained since the onset of the pandemic, many of the precise mechanisms underlying SARS-CoV-2 pathogenesis remain incompletely understood. Continued investigation into these processes is not only essential for advancing our biological understanding of viral–host interactions but also remains a critical public health priority, as emerging variants and long-term sequelae continue to challenge healthcare systems worldwide.

SARS-CoV-2 is a positive-sense single-stranded RNA virus whose genome encodes structural, non-structural, and accessory proteins that orchestrate viral entry, replication, and immune evasion [2,3,4]. The viral particle contains four major structural proteins: spike (S), envelope (E), membrane (M), and nucleocapsid (N). Viral entry is primarily mediated through the interaction of the spike (S) protein with the host ACE2 receptor, a process facilitated by cellular proteases such as TMPRSS2 [5,6]. In addition to mediating viral entry, structural proteins also contribute to host-cell remodeling and immune evasion. The envelope (E) and membrane (M) proteins participate in viral assembly and release, while the M protein has also been implicated in suppression of antiviral signaling through interactions with MAVS and TBK1. The nucleocapsid (N) protein encapsulates the viral RNA genome and interferes with RIG-I activation through disruption of TRIM25-mediated signaling [7].

Beyond the structural proteins, the SARS-CoV-2 genome encodes sixteen non-structural proteins (Nsps1–16), which regulate viral replication, RNA processing, host translational shutdown, and innate immune modulation, as well as multiple accessory proteins, including ORF3a, ORF3b, ORF3c, ORF6, ORF7a, ORF7b, ORF8, ORF9b, ORF9c, and ORF10 [8]. Although accessory proteins are not essential for viral replication in vitro, they play important roles in viral pathogenicity and immune escape. Several of these proteins directly target mitochondrial and interferon-associated pathways. For example, ORF6 blocks nuclear translocation of STAT1 and IRF3, ORF3a modulates apoptosis and autophagy, and ORF9b localizes to the mitochondrial membrane, where it interacts with the Tom70 receptor to suppress antiviral signaling cascades [9].

Following viral entry, the viral RNA is recognized by RIG-I-like receptors, initiating an innate antiviral immune response. This process involves the mitochondrial antiviral-signaling protein MAVS, which activates downstream signaling pathways including TBK1, IRF3/7, and NF-κB, culminating in the expression of interferon-stimulated genes (*ISGs*) [10,11,12]. However, SARS-CoV-2 proteins can actively subvert these pathways, contributing to an imbalanced antiviral response. In this context, mitochondria act as central hubs linking metabolism, innate immunity, and cell fate during SARS-CoV-2 pulmonary infection [13]. Alterations in mitochondrial dynamics—including shifts between fission and fusion—can influence both antiviral defense and viral replication through modulation of bioenergetics, mitophagy, reactive oxygen species (ROS) production, and metabolic flexibility [13,14,15].

This mitochondrial regulatory axis is particularly relevant during SARS-CoV-2 infection, as several viral proteins, including M, ORF3a, ORF9b, and spike, have been reported to disrupt mitochondrial homeostasis. These disruptions result in decreased mitochondrial membrane potential (ΔΨm), impaired oxidative phosphorylation (OXPHOS), increased reactive oxygen species (ROS) production, and induction of apoptosis [14,16,17].

Mitochondria are highly dynamic organelles that undergo continuous cycles of fission and fusion to regulate cellular morphology, metabolism, quality control, and survival. Increasing evidence indicates that SARS-CoV-2 directly interferes with these processes in order to suppress host antiviral responses and promote viral persistence and replication [18,19].

Mitochondrial fusion is mediated by the mitofusins MFN1 and MFN2, located on the outer mitochondrial membrane, together with OPA1 on the inner mitochondrial membrane. Fusion promotes the exchange of mitochondrial contents and contributes to the maintenance of mitochondrial integrity under stress conditions. In contrast, mitochondrial fission is primarily mediated by the cytosolic GTPase DRP1, which is recruited to the outer mitochondrial membrane to constrict and divide mitochondria [18,20].

Furthermore, SARS-CoV-2 infection has been associated with metabolic reprogramming, increased production of reactive oxygen species (ROS), mitochondrial stress, and inflammatory amplification, contributing to endothelial dysfunction and altered cellular homeostasis [14,15,21].

Recent studies have also identified ISG15 as an important regulator of mitochondrial dynamics and homeostasis. In addition, mitochondrial proteins have been identified among potential ISGylation targets, supporting a broader role for ISG15 in mitochondrial quality control and stress adaptation [22,23].

In addition to morphological remodeling, post-translational modifications (PTMs) such as ISGylation and SUMOylation provide a further regulatory layer. ISGylation of DRP1 by interferon-stimulated gene 15 (ISG15), mediated by the E3 ligase HERC5, promotes mitochondrial fission, while de-ISGylation shifts the balance toward hyperfusion; SARS-CoV-2 PLpro has been shown to act as a deISGylase, directly interfering with this process [24]. Conversely, SUMOylation regulates DRP1 activity, and reduced SUMO1/2 expression could destabilize fission/fusion control and mitochondrial quality regulation [25].

Among the ISGs, ISG15 plays a pivotal role in both antiviral immunity and mitochondrial regulation [26]. ISG15 is involved in ISGylation, a post-translational modification that targets proteins associated with mitochondrial dynamics (e.g., DRP1, OPA1, and MFN1/2), mitochondrial DNA replication, and assembly of the respiratory chain [27,28,29,30]. These functions highlight ISG15 as a key mediator of mitochondrial homeostasis during viral infection.

Furthermore, the viral papain-like protease (PLpro) antagonizes the ISG15 system by cleaving ISG15 from its targets, thereby impairing mitochondrial function and modulating the interferon response [31]. In its free, unconjugated form, ISG15 can also act as a cytokine, influencing immune cell behavior [31,32,33,34,35].

Given the central role of mitochondria in antiviral defense and cellular metabolism [36], in this study, we focused on the hypothesis that SARS-CoV-2 infection promotes a coordinated remodeling of mitochondrial homeostasis and antiviral signaling in LC-HK2 cells, involving changes in mitochondrial dynamics and post-translational regulatory pathways. Understanding how mitochondrial pathways are altered during SARS-CoV-2 infection is crucial. In this study, we focused on the hypothesis that SARS-CoV-2 infection promotes a coordinated remodeling of mitochondrial homeostasis and antiviral signaling in LC-HK2 cells, involving changes in mitochondrial dynamics and post-translational regulatory pathways. To address this, we conducted a global proteomic analysis of SARS-CoV-2–infected human non-small cell lung carcinoma (LC-HK2) cells using mass spectrometry and reanalyzed publicly available proteomics datasets to identify key pathways associated with viral infection, mitochondrial stress, and immune regulation. The proteomic findings were used to guide a targeted experimental workflow aimed at evaluating whether mitochondrial alterations and antiviral post-translational regulatory processes occur concomitantly within the same cellular system in a temporally regulated manner. These findings guided subsequent analysis using confocal microscopy, high-content imaging, and RT-qPCR to characterize mitochondrial morphology, fission/fusion-associated markers, and their interplay with ISG15 and SUMO 1 and 2/3. Through this combined strategy, we showed that SARS-CoV-2 infection is associated with altered mitochondrial dynamics, changes in antiviral signaling, and differential mitochondrial localization of ISG15 and SUMO 2/3-related signals. Together, our study provides an integrated view of how SARS-CoV-2 infection affects the mitochondrial–antiviral regulatory axis in LC-HK2 cells, highlighting mitochondrial remodeling and ISG15/SUMO-associated regulation as the central focus of the work.

## 2. Materials and Methods

**Figure 1:**
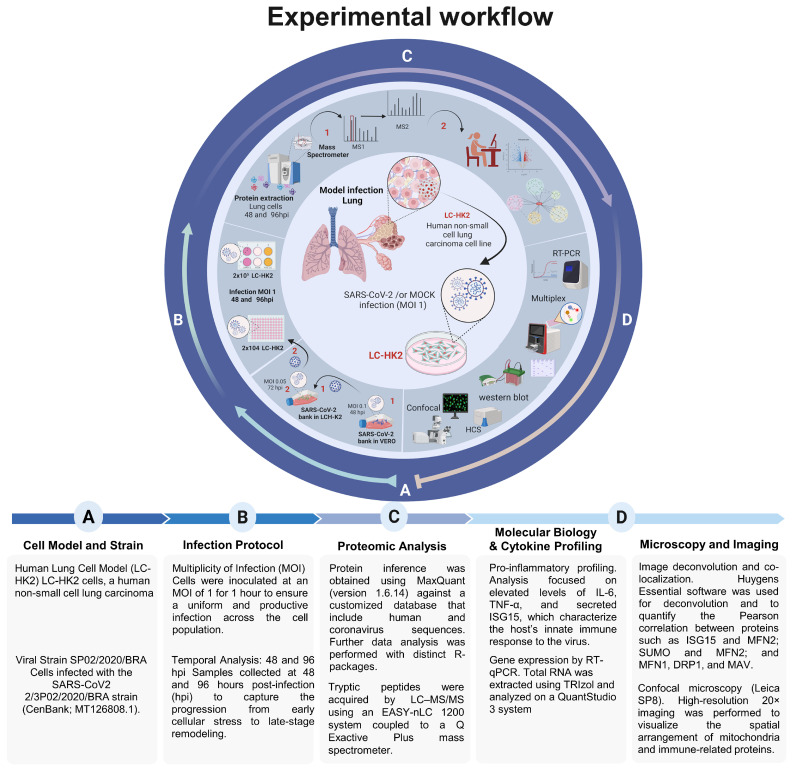
Experimental workflow. Schematic representation of the experimental design and methodologies employed in this study, including cell culture, sample processing, and downstream analytical techniques. Created in BioRender. Zambelli, V. (2026) https://BioRender.com/hmwkoiz (accessed on 11 April 2026).

To provide an overview of the experimental strategy, Figure 1 summarizes the workflow used in this study, including cell culture conditions, sample processing steps, and the downstream analytical methodologies applied.

### 2.1. Cell Culture and Work Seed Virus Bank Production

The SARS-CoV-2 strain used in this work (SARS-CoV-2/SP02/2020/BRA virus; GenBank accession number: MT126808.1 B.1.1.28) was kindly provided by Dr. Edison Luis Durigon (Brazilian virus network, Institute of Biomedical Science, University of São Paulo) [37]. The Working Virus Seed Stock (WVSS) was prepared using Vero CCL-81.4 cells (ATCC, Manassas, VA, USA) [38]. The WVSS was introduced into LC-HK2 cells (initially called HK2, a cell line established from human non-small cell lung carcinoma) [39] to study the spread of the virus in lung cells. These cells were generously provided by Dra. Glaucia Santelli (Department of Cell and Developmental Biology, Institute of Biomedical Sciences, University of São Paulo). For research purposes, the lung cells were grown in a basal medium, Dulbecco’s modified Eagle’s medium (DMEM)-F12 (11320033, Gibco, Thermo Fisher Scientific. Grand Island, NY, USA), supplemented with 10% fetal bovine serum (FBS; 12657029, Gibco- Thermo Fisher Scientific, Grand Island, NY, USA). The cells were then infected at a multiplicity of infection (MOI) of 0.05 and incubated for 72 h, at 37 °C, in a 5% CO_2_-containing atmosphere. The virus suspension was collected, cleared of cellular debris by means of centrifugation, and filter-sterilized (0.22 µm), aliquoted, and stored at −80 °C in sucrose, sodium phosphate, and glucose preservation solution (7.462 g/L sucrose, 0.0517 g/L KH_2_PO_4_, 0.1643 g/L K_2_HPO_4_·3H_2_O, and 0.0907 g/L potassium glutamate). The virus titers were determined in terms of the 50% tissue culture infectious dose (TCID_50_) [38,40]. All viral manipulations were conducted in compliance with the WHO guidelines for handling SARS-CoV-2 specimens, in a Biosafety Level-3 facility.

### 2.2. Inoculation of LC-HK2 with SARS-CoV-2

The LC-HK2 cells used in this study have previously been used in numerous studies [39,41,42,43,44,45]. LC-HK2 cells were selected because they provide a lung-derived epithelial model with strong adherence, a well-organized cytoskeleton, and suitability for high-resolution imaging and mitochondrial network analysis [41,44]. Compared with commonly used lung-derived models such as A549 and Calu-3, LC-HK2 cells exhibit prominent actin organization and focal adhesion structures, features relevant to investigating virus-induced cellular remodeling and intracellular trafficking [44]. LC-HK2 cells also display high proliferative and stress-tolerant capacity, supporting experimental reproducibility and facilitating longitudinal analyses at later stages of SARS-CoV-2 infection. The cells were grown, as mentioned previously, until they reached 80% confluence, to establish the cell culture. All cell cultures were maintained at 37 °C in an atmosphere with 95% humidity and 5% CO_2_. The cells were subcultured by means of enzymatic digestion with trypsin-EDTA (0.05%) containing Phenol Red (25300054, Gibco, Thermo Fisher Scientific, Grand Island, NY, USA). LC-HK2 cells were plated in 6-well plates at a density of 2 × 10^5^ cells/mL or in 96-well plates at a density of 2 × 10^4^ cells/mL, for 24 h, and then infected. LC-HK2 cells were infected with SARS-CoV-2, the WVSS for which was previously grown in lung cells. The cells were infected at an MOI of 1 for 1 h and then maintained in basal growth media (DMEM-F12 with 10% FBS) for 48 and 96 h post-infection (hpi). As a negative control, some cells were treated with mock medium for the same duration. The impact of the virus on cellular pathology was observed over a period of 96 h. To conduct cytokine and proteomic analyses, outside the NB3 facility, the cell and supernatant samples were either inactivated using gamma radiation, at a dose of 15 kGy [38,46,47] or fixed in 4% paraformaldehyde for 1 h, for immunofluorescence analysis. Alternatively, the cells were collected using TRIzol™ (15596026, Thermo Fisher Scientific, Carlsbad, CA, USA) for molecular biology methods [47]. The plates that had been exposed to viral infections were sealed, treated with ultraviolet light, and then removed from the NB3 facility for further analysis.

### 2.3. Sample Preparation and Liquid Chromatography Tandem Mass Spectrometry (LC-MS/MS) Analysis

The supernatants were removed from the cell culture flasks at 48 and 96 hpi, and the cells were rinsed three times with phosphate buffer saline (PBS) to remove the growth media. As previously described [47], the cells were collected in 1 mL of a solution containing 2 M urea and 5 mM Dithiothreitol (DTT) and then stored at −80 °C until protein extraction. The cells were lysed while on ice and underwent ultrasonication (5 cycles: 30 s of sonication followed by 30 s on ice) before being centrifuged at 15,000× *g* for 30 min, to remove any insoluble material. The sample supernatants were subjected to the Fast and Secure Protocol [48,49] using Microcon^®^ 10 kDa centrifugal filter units (MRCPRT010, Merck Millipore, Carrigtwohill, Co. Cork, Ireland). After trypsin digestion in the filters, the resulting peptides were desalted using in-house prepared StageTips containing SDB-XC membranes and subsequently dried under vacuum in a centrifugal concentrator (22331, Eppendorf, Hamburg, Germany). Peptides (250 ng) were resuspended in 20 µL of 0.1% formic acid and analyzed by LC-MS/MS using an EASY-nLC™ 1200 system (LC-030378, Thermo Fisher Scientific, Waltham, MA, USA) coupled to a Q Exactive™ Plus mass spectrometer (03893L, Thermo Fisher Scientific, Waltham, MA, USA) at the Mass Spectrometry Unit of the Centre of Excellence in New Target Discovery (CENTD), Butantan Institute, São Paulo, SP, Brazil. For each sample, a mixture of peptides was loaded onto an Acclaim™ PepMap™ 100 C18 Trap Column (Thermo Fisher Scientific, Waltham, MA, USA). This column had a particle size of 3 μm, pore size of 100 Å, internal diameter of 75 μm, and length of 20 mm. The column was connected to an analytical column Acclaim™ PepMap™, which had a particle size of 2 µm, pore size of 100 Å, length of 150 mm, and inner diameter of 50 µm. The flow rate was set to 200 nL/min. The mobile phases used were solvent A = 0.1% *v*/*v* formic acid and solvent B = 80% *v*/*v* acetonitrile and 0.1% *v*/*v* formic acid. A linear gradient was used, starting at 5% B and increasing to 30% B in 50 min, then to 60% B in 13 min, and then to 100% B in 2 min. The final step was a 5 min phase of 100% B. The chromatographic run ended with a 5 min step at 100% solvent. Mass spectrometry data were acquired in positive ionization and data-dependent acquisition mode. Full MS spectra were collected over an *m*/*z* range of 300–1500, and the seven most abundant precursor ions were subsequently selected for MS/MS fragmentation by higher-energy collisional dissociation (HCD). The isolation window was set to 2.0 *m*/*z*, and dynamic exclusion was applied for 60 s. The target automatic gain control value and maximum injection time were set to 3 × 10^6^ and 200 ms, respectively, for the survey MS scan, and 2 × 10^5^ and 120 ms, respectively, for the fragmentation ion scan (MS/MS). All analyses were conducted in biological duplicates and experimental triplicates.

### 2.4. Proteomics Data Processing and Comparative Proteomic Analysis

The analysis of MS and MS/MS spectra was carried out using MaxQuant version 1.6.14 [50]. The searches were conducted against a customized database that included all coronavirus sequences, reviewed *Homo sapiens* sequences downloaded from UniProt (total of 20,845 sequences, downloaded on 10 January 2020), and common MS contaminants. The Andromeda search engine was configured to detect specific tryptic peptides at a false discovery rate (FDR) of 0.01, utilizing a decoy database. It also performed label-free quantification with default parameters. The search parameters included variable modifications for methionine oxidation and acetylation of protein N-termini, and a fixed modification for carbamidomethylation of cysteine. The identified and quantified protein groups underwent post-processing in the R programming environment. Proteins identified as contaminants, as reverse sequences, or only by site were filtered out. To analyze protein significance, the data were input into the Limma R 3.62.2 package [51]. Proteins showing *p*-values < 0.05 and fold change (FC) values ≥ 1.5 (−0.585 < log2FC > 0.585) were classified as differentially abundant between SARS-CoV-2–infected and mock-infected samples. Functional enrichment analysis was subsequently performed using the Reactome database, considering pathways with *p*-values < 0.05 [52], and protein–protein interaction network analysis was performed using STRING [53]. Network enrichment analysis was performed using the ClueGO v2.5.10 [54] and AutoAnnotate v1.5.2 [54] plugins in Cytoscape software 3.10.3 [55]. All biological processes and pathways enriched from both the Gene Ontology (GO) and Reactome databases showed a corrected *p* < 0.05, which were determined using the hypergeometric test and Bonferroni step-down correction for multiple hypothesis testing.

To compare our proteomics dataset with previously published studies, we screened the literature for papers reporting proteomic analyses of SARS-CoV-2–infected lung cells. Studies lacking accessible supplementary (Table S1) data with processed proteomics results were excluded. In total, 6 studies were included in our analysis: Crozier et al., 2022; Babačić et al., 2023; Puray-Chavez et al., 2021; Stukalov et al., 2021; Grossegesse et al., 2022; Hatton et al., 2021 [56,57,58,59,60,61]. Differences in experimental design, such as cell lineages, multiplicity of infection, and quantification strategies, are summarized in Supplementary Table S1. Because the studies employed different quantification approaches (e.g., label-free methods and tandem mass tags), we relied on the statistical results and significance cutoffs reported in each paper, specifically the comparisons between mock and SARS-CoV-2–infected cells. Using the lists of differentially expressed proteins provided by each study, we constructed an UpSet plot to examine unique and shared targets. Proteins identified as differentially expressed in at least four studies were submitted to STRING for enrichment analysis.

We also generated a correlation network to investigate how proteins shared between our dataset and others behave in terms of up- or downregulation in our study. To do this, we filtered our dataset for proteins classified as differentially regulated both in our analysis and in at least one additional study, and calculated Pearson correlation coefficients using the log2-transformed intensities of each sample. Protein pairs with correlation coefficients >0.7 or <−0.7 were retained. The network, built in R using the corrr package, represents differentially regulated proteins as nodes, with edges depicting their correlation relationships. Finally, to complement this analysis, we compiled a dataset containing the fold changes of mitochondrial proteins reported across studies. Key regulators of mitochondrial metabolism, fusion, and fission were selected according to Gene Ontology annotation and literature reports, and their fold changes were visualized using heatmaps.

### 2.5. Gene Expression Analysis

LC-HK2 cells were infected with SARS-CoV-2, and at 48 and 96 hpi, the supernatants were removed from these cultures, the cells were washed with PBS, and TRIzol™ (15596018, Invitrogen Thermo Fisher Scientific, Carlsbad, CA, USA) was added to each well. The lysates were stored at −80 °C until RNA extraction. Total RNA was isolated and purified using the Illustra™ RNAspin Mini Kit (25-0500-72, GE Healthcare, Freiburg, Germany), as per the manufacturer’s protocol. The amount of total RNA was measured using a NanoDrop^®^ ND-1000 spectrophotometer (5225, Thermo Scientific, Wilmington, DE, USA). Next, cDNA was synthesized using 500 ng of RNA template, oligo-dT, and random hexamer primers, according to the SuperScript™ III First-Strand Synthesis SuperMix (18080-400, Invitrogen, Thermo Fisher Scientific, Carlsbad, CA, USA). Gene-specific primers and the Fast SYBR™ Green Master Mix (4385612, Applied Biosystems, Thermo Fisher Scientific, Vilnius, Lithuania) were used for the real-time quantitative polymerase chain reaction (RT-qPCR), which was performed on a QuantStudio™ 3 Real-Time PCR System (A28131, Thermo Fisher Scientific, Woodlands, Singapore). The RT-qPCR program consisted of 40 cycles at 95 °C for 15 s followed by 60 °C for 1 min. The mRNA expression levels were normalized to the mean Ct for the ribosomal protein L37a (RPL37A), which served as an endogenous control. The data were analyzed using the delta Ct method (Pfaffl, 2001) [62] and can be found in Supplementary Table S2. The methodologies used in this study were performed following the same experimental procedures previously described by T.C. Melo et al. (2022) [47].

### 2.6. Indirect Immunofluorescence, Deconvolution, Colocalization, Puncta Quantification, and 3D Reconstruction

LC-KH2 cells were placed in a 10-well Cellview cell culture slide, PS, 75/25 MM (543079) or 96-well Microplate, PS, F-Bottom, µClear^®^ (655986) (both from Greiner Bio-One GmbH, Baden-Württemberg, Germany), at a density of 1 × 10^4^ cells/cm^2^. The cells were cultivated for 2 d in the medium and culture conditions mentioned previously and then inoculated with SARS-CoV-2 (at a MOI of 1) or mock medium for 1 h, to allow for adsorption. Following that, the supernatant was removed, and DMEM-F12 was added to the culture. At 48 or 96 hpi, the cells were fixed with 4% paraformaldehyde for 30 min at 4 °C and then washed twice with 1× PBS. A 0.1% Triton™ X-100 (X100-500ML, Sigma-Aldrich, St. Louis, MO, USA) solution was used to permeabilize the cells for 15 min, at 4 °C, following which the cells were washed twice with 1× PBS. Bovine serum albumin 5% (BSA; A7906-50G, Sigma-Aldrich, St. Louis, MO, USA) was used to block the cells at room temperature for 40 min, following which they were exposed to a primary antibody overnight. All primary and secondary antibodies used in this study, together with their respective sources, catalog numbers, and working dilutions, are detailed in Supplementary Table S3. After antibody incubation, cells were washed with 1xPBS and nuclei were counterstained with Hoechst 33342 0.1 mg/mL; (H3570, Invitrogen, Thermo Fisher Scientific, Eugene, OR, USA). The cells were then scanned along the x, y, and z axes with an SP8 confocal microscope (Leica Microsystems, Hesse, Germany), using a 20×/0.75NA or 63×/1.4NA objective lens. Laser excitation was performed at the wavelengths of 405, 488, and 638 nm, and the LAS X software 3.5 (Leica Microsystems, Hesse, Germany) was utilized for acquisition. The Huygens Essential software 25.01 (Scientific Volume Imaging, Hilversum, North Holland, The Netherlands, http://svi.nl/ accessed on 8 May 2025) was used to image deconvolution for colocalization of ISG15 and MFN2 proteins, as well as SUMO2/3 and MFN1, MFN2, DRP1, and MAVs. Additionally, CellProfiler™ software, version 4.2.5 (www.cellprofiler.org/ accessed on 11 April 2025), was used to quantify the number of puncta and determine the cellular localization of proteins (Supplementary Figure S1). Immunofluorescence was assessed in three independent experiments, and data were compared using the Mann–Whitney U test, on Prism version 10 GraphPad (GraphPad Software, LLC, Boston, MA, USA. Data are represented as mean ± standard deviation. Statistical significance was considered at a *p*-value of less than 0.05. Perinuclear mitochondrial localization was assessed based on radial fluorescence intensity. Fluorescence was quantified using a 12-bin concentric segmentation approach. Mean values per bin were averaged across cells and interpolated to generate continuous spatial maps. Topographic contour plots were used to visualize radial distribution patterns. All analyses were performed in R using the packages ggplot2, dplyr, readxl, and patchwork.

### 2.7. Cytokine Profiling in Lung Cells Infected with SARS-CoV-2

Cell-free supernatants were collected from LC-HK2 cells infected with SARS-CoV-2 for cytokine secretion analysis at 48 and 96 hpi. Cytokine quantification was conducted by means of multiplex analysis, using the Milliplex^®^ MAP Human Cytokine/Chemokine Magnetic Bead Panel, IL-10, IL-1β, IL-4, IL-6, IL-8, IL18, IP10, TNFɑ, and IFN ɑ2 (HCYTOMAG-60K and HSTCMAG-28SK, EMD Millipore Corporation, Burlington, MA, USA), as per the manufacturer’s recommendations. Data were acquired using the Luminex™ 200 system and xPONENT^®^ software, version 4.3 (Luminex Corporation, Austin, TX, USA) and analyzed using Milliplex^®^ Analyst 5.1. Cytokine or chemokine detection was carried out in the pg/mL range. All analyses involve biological triplicates and experimental duplicates, with cytokine release-inducing treatments, such as interleukin (IL)-1β (1 ng/mL) and polyinosinic/polycytidylic acid [Poly (I:C)] (10 μg/mL), used as internal controls of the experiment.

For Western blot analysis of conditioned medium, experiments were performed using biological triplicates with experimental duplicates. Due to the limited volume of conditioned medium obtained from each sample and the low abundance of extracellular proteins, conditioned media from biological replicates belonging to the same experimental condition were pooled and subsequently concentrated prior to SDS-PAGE and immunoblotting. Therefore, each lane represents an independent experiment of pooled biological triplicates of the sample condition. Western blot results from conditioned medium were interpreted as representative/semi-quantitative detection of SARS-CoV-2 S protein and ISG15 in extracellular fractions.

### 2.8. Use of AI

Generative AI tools were used in a limited manner, mostly for language refinement. NotebookLM Standard, free web-based version (Google, Mountain View, CA, USA; https://notebooklm.google/ accessed on 6 March 2026) was used to assist in the conceptual organization and drafting of the graphical abstract. The experimental workflow figure was created using BioRender.com, based on the authors’ predefined study design. ChatGPT-5 (OpenAI) and Gemini (Google, Mountain View, CA, USA; https://gemini.google.com/ accessed on 20 February 2026) were used for linguistic refinement, including grammar correction, clarity improvement, and reducing redundancy. Scite was used to support literature identification and cross-referencing.

All AI-assisted outputs were critically reviewed and edited by the authors. No AI tools were used for data analysis, interpretation of results, or generation of scientific conclusions. The authors take full responsibility for the accuracy and integrity of the work.

## 3. Results

### 3.1. Infection of Lung Cells (LC-HK2) with SARS-CoV-2

**Figure 2:**
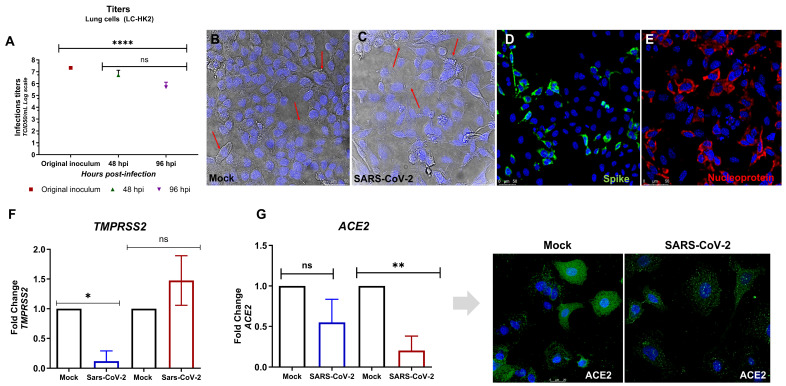
Productive infection of LC-HK2 with SARS-CoV-2. LC-HK2 cells were infected with SARS-CoV-2 at a multiplicity of infection of 1 or mock for 1 h and then cultured for up to 48 or 96 h. (**A**) The infectious titer of the virus, as measured using the TCID50 assay at 48 and 96 hpi, showed viral replication. The values are represented as mean ± standard deviation of three independent biological samples. (**B**,**C**) Confocal microscopy images showing the cytopathic effects in the cytoskeleton, including cytoplasmic extensions in the infected LC-HK2 cells, as compared to those in the mock control, at 48 hpi. The extensions are highlighted using red arrows. (**D**,**E**) Confocal microscopy images showing positive immunostaining for SARS-CoV-2 S and N proteins in LC-HK2 cells infected at a multiplicity of infection of 1. (**F**,**G**) Quantification of the RT-qPCR analysis in terms of fold-change values showed a decrease in the mRNA expression of *TMPRSS2* at 48 hpi and that of *ACE2* at 96 hpi. The values represent data from three independent experiments, which revealed a significant reduction (*p* = 0.0015). Asterisks indicate statistically significant differences, as determined using GraphPad Prism, with *p* < 0.05 considered statistically significant: * *p* < 0.05, ** *p* < 0.01, and **** *p* < 0.0001. Immunofluorescence analysis of the expression of *ACE2* at 96 hpi. Green: SARS-CoV-2 S antibody indirectly labeled with the Alexa Fluor™ 488-conjugated anti-rabbit secondary antibody; red: recombinant SARS-CoV-2 *N* protein antibody indirectly labeled with propidium iodide-conjugated anti-human IgG secondary antibody; blue: nuclei stained with Hoechst 33342. TCID50, 50% tissue culture infectious dose; hpi, hours post-infection; SARS-CoV-2, severe acute respiratory syndrome coronavirus 2; *S*, spike protein; *N*, nucleocapsid protein; RT-qPCR, real-time quantitative polymerase chain reaction.

To confirm the infection of the human non-small cell lung carcinoma cells (LC-HK2) with SARS-CoV-2, the cells inoculated with the virus at an MOI of 1 were subjected to viral titer measurements, cell morphology analysis, and fluorescence staining for two specific virus proteins, at 48 and 96 hpi. The infectious titer was found to be maintained over the evaluated time points, as measured by means of the TCID50 assay (Figure 2A), and virus-induced cytopathic effects were observed even as early as 48 hpi, manifested as rounding of the infected cells, vacuolization, and the appearance of cytoplasmic or nuclear inclusion bodies (Figure 2B,C); these results indicated productive infection. Immunofluorescence imaging analysis showed that LC-HK2 cells were positive for S and N proteins, thus confirming virus infection (Figure 2D,E). Upon evaluation of important cellular receptors for SARS-CoV-2 infection, positive RNA levels of *TMPRSS2* and *ACE2* were observed in the infected LC-HCK2 cells, at 48 and 96 hpi. Compared to the mock group, there was a slight decrease in *TMPRSS2* expression at 48 hpi (Figure 2F) and a 50% decrease in *ACE2* expression at 96 hpi (Figure 2G). In addition, immunofluorescence analysis showed lesser staining for ACE2 in the infected cells, as compared to that in the mock cells, indicating a decrease in its protein expression at 96 hpi (Figure 2G).

### 3.2. Characterization of the Proteome in SARS-CoV-2–Infected Cells and Comparative Analysis with Published Studies

**Figure 3:**
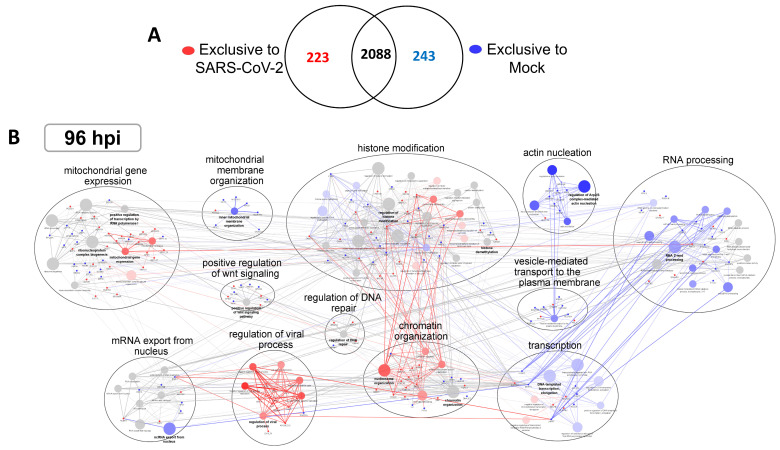
Biological processes and pathways of exclusive proteins enriched in LC-HK2 cells, at 96 hpi. (**A**) A Venn diagram shows the number of proteins in cells infected with SARS-CoV-2 and mock, at 96 hpi. Proteins in LC-HK2 infected with SARS-CoV-2 were identified using global proteome analysis. Only proteins exclusively identified in mock or infected conditions were analyzed using PEAKS Studio X software. (Bioinformatics Solutions Inc., Waterloo, ON, Canada.); (**B**) Enriched Reactome pathways and biological processes (GO) of proteins identified exclusively in mock-infected (blue) and SARS-CoV-2–infected (red) LCH-K2 cells. Nodes represent enriched terms for biological pathways or processes. The dots represent the main proteins associated with each biological process or pathway, while the connections between the nodes indicate the shared proteins between them. The significance of terms with a corrected *p*-value < 0.05 (as evaluated using a right-sided hypergeometric test with FDR Bonferroni correction) was directly proportional to the size of the nodes. AutoAnnotate was used to cluster and identify biological processes (GO), and the enriched Reactome pathways are indicated by means of black circles. The pathways enriched in the infected cells included mitochondrial gene expression, mitochondrial membrane organization, regulation of viral processes, chromatin organization, and vesicle-mediated transport to the plasma membrane.

As SARS-CoV-2–infected LC-HK2 cells had not yet been characterized at the proteome level, we first performed an unbiased mass spectrometry-based proteomic screening to define infection-associated changes in this cellular model and to identify pathways for subsequent targeted validation. For comparison of the global proteome of the mock and infected LC-HK2, the cells were collected at 48 and 96 hpi and processed for MS analyses. Initially, the proteins exclusively identified in each group (mock or infected cells) were evaluated. At 48 hpi, a total of 2660 protein groups were identified in the infected LC-HK2 cells, while 2668 were identified in the mock cells; among these, 246 and 254 proteins were found exclusively in the infected and mock groups, respectively (Supplementary Table S4 and Figure S2). At 96 hpi, a total of 2331 proteins were identified in the mock group, while 2311 were identified in the infected cells; of these, 223 and 243 protein groups were present exclusively in the infected and mock groups, respectively (Figure 3A).

Among the proteins identified only in the infected cells, eight SARS-CoV-2 proteins were identified at 48 hpi: N, S, M, replicase polyprotein 1ab (pp1ab), ORF3a, ORF7a, ORF8, and ORF9b; four viral proteins were identified at 96 hpi: N, S, M, and pp1ab (Supplementary Table S4). Differences in viral protein detection between time points should be interpreted within the limitations of the approach, as data-dependent acquisition-based proteomics in a highly abundant host background may limit detection of low-abundance viral peptides. In addition, later infection stages associated with cellular stress and cytopathic effects may reduce viral protein production and detectability. The GO enrichment analysis of proteins exclusive to each group showed a distinct pattern of enriched terms (Figure 3B). The enriched terms for proteins exclusively found in the infected cells were related to the regulation of the virus process, chromatin organization, histone modification, and mitochondrial gene expression (Figure 3B). These results further validated the occurrence of productive infection of LC-HK2 cells.

**Figure 4:**
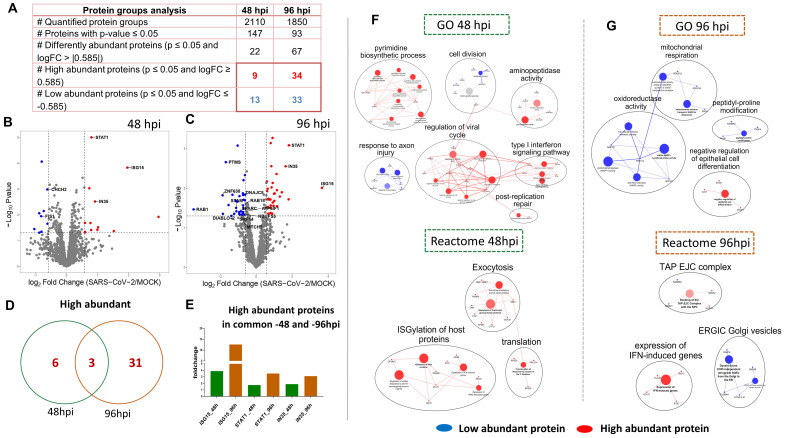
Label-free relative quantitative proteomic analysis of LCH-K2 cells in response to SARS-CoV-2 infection. (**A**) Protein groups and differentially abundant proteins identified upon comparison of the SARS-CoV-2–infected and mock-infected LC-HK2 cells, at 48 and 96 hpi, with the number of high-abundance proteins indicated in red and the number of low-abundance proteins indicated in blue. (**B**,**C**) Volcano plots of the relatively quantified protein groups, showing high abundance proteins in red (log2FC ≥ 0.58 and –log10 *p* > 1.12 = *p* < 0.05) and low abundance proteins in blue (log2FC ≤ −0.58 and −log10 *p* > 1.12 = *p* < 0.05). Non-differentially abundant proteins are represented in gray. (**D**,**E**) At both the time points of 48 and 96 hpi, three proteins (ISG15, STAT1, and IN35) showed high abundance (FC ≥ 1.5×), which increased over the time of infection. (**F**,**G**) Network representation of the enriched terms for up (red) or down (blue) regulated protein clusters in SARS-CoV-2–infected LC-HK2 samples, at 48 or 96 hpi, respectively. Nodes depict the enriched pathway, and biological process terms were assigned according to Reactome and Gene Ontology (GO) annotations. Connections between nodes indicate shared proteins among pathways or processes, while dots represent the principal proteins associated with each term. Node size corresponds to the relative enrichment or number of associated proteins (corrected *p* < 0.05, as assessed using a right-sided hypergeometric test with Bonferroni step-down FDR correction). AutoAnnotate was used to cluster and label related biological processes (GO) or Reactome pathways. At 48 hpi, the enriched terms included regulation of the viral cycle, type I IFN signaling, and ISGylation of host proteins, which have been highlighted with black circles. At 96 hpi, the enriched terms included regulation of the expression of IFN-induced TAP-EJC (required by the Nuclear Pore Complex for transport of mRNA from the nucleus to the cytoplasm), in addition to mitochondrial respiration, oxidoreductase activity, and ERGI Golgi vesicles (retrograde traffic from the cis-Golgi to the ERGI). hpi, hours post-infection; IFN, interferon; SARS-CoV-2, severe acute respiratory syndrome coronavirus 2; FC, fold-change; GO, Gene Ontology; IFN, interferon.

Furthermore, we performed a label-free relative quantitative comparison of the proteins in the infected and mock groups. A total of 2110 and 1850 proteins were relatively quantified at 48 and 96 hpi, respectively (Supplementary Tables S5 and S6). At 48 and 96 hpi, 147 (Figure 4A,B) and 93 proteins (Figure 4A,C), respectively, were found to be differentially abundant. Among the proteins that were found to be in lower abundance in the infected cells at 48 hpi, we highlight two that are involved in mitochondrial dynamics: coiled-coil-helix-coiled-coil-helix domain-containing protein 2 (CHCHD2) and mitochondrial fission protein 1 (FIS1) (Figure 4B). At 96 hpi, we identified four proteins involved in distinct mitochondrial processes: ATP synthase inhibitory factor subunit 1 (ATP5IF1), NADH dehydrogenase [ubiquinone] iron–sulfur protein 5 (NDUFS5), mitochondrial carrier homolog 2 (MTCH2), and mitochondrial diablo homolog (DIABLO-2). Comparison of the differentially abundant proteins at 48 and 96 hpi also led to the identification of three common proteins that were in higher abundance in the infected cells and which showed an increase in abundance over time: ISG15, STAT1, and IN35/IFI35 (Figure 4D,E). These proteins are known to be involved in numerous immune system pathways related to IFN signaling. GO enrichment analysis and the Reactome gene annotation terms showed that the downregulated proteins were mostly associated with metabolic processes, such as mitochondrial respiration and oxidoreductase activity, while the upregulated proteins were associated with antiviral mechanisms, IFN signaling, and mRNA transport, at both time points (Figure 4F,G).

**Figure 5:**
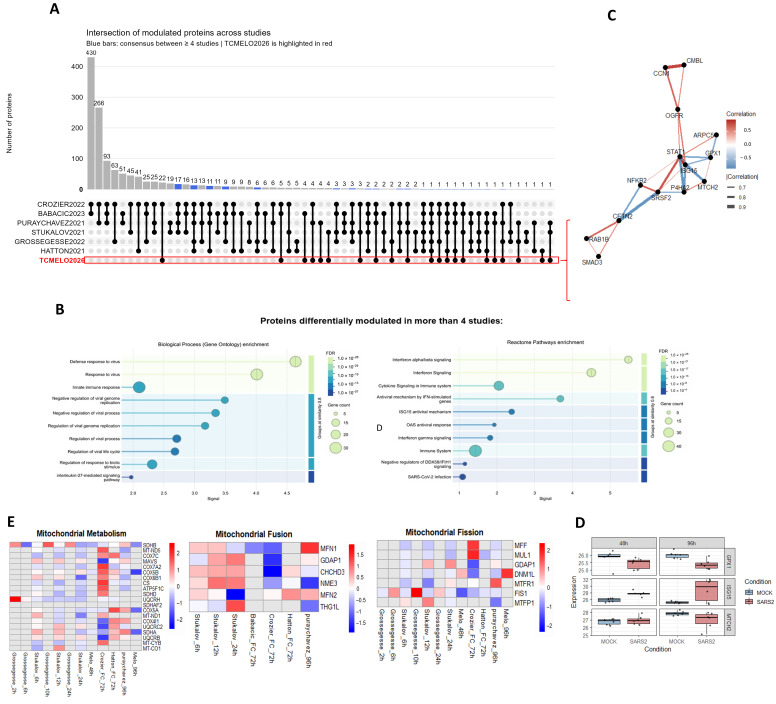
Comparative proteomic mapping of mitochondrial-related proteins in SARS-CoV-2–infected pulmonary epithelial cells. (**A**) The red highlight indicates proteins shared between the dataset from this study (TC Melo et al.) and publicly available dataset generated lung-derived cell models. were compared with publicly available datasets from other studies using lung-derived cell models. The UpSet plot displays the intersections of differentially regulated proteins across the studies included in our analysis. The set size represents the number of proteins identified as differentially expressed in each study, while the intersection size indicates the number of shared differentially expressed proteins between studies. (**B**) Enrichment analysis was performed in STRING using proteins that were differentially modulated in at least four independent studies, revealing Gene Ontology biological processes and Reactome pathways related to antiviral defense mechanisms, ISG15 conjugation, and interferon signaling. (**C**) The correlation network highlights overlapping proteins between our dataset and other studies. This set includes MTCH2, GPX1, ISG15, STAT1, NFKB2, SMAD3, ARPC5, CETN2, RAB1B, CCN1, and P4HA2—key nodes linking mitochondrial remodeling, interferon signaling, and cytoskeletal regulation. Blue and red lines represent negative and positive correlations, respectively. Line width is proportional to the strength of the correlation. (**D**) Boxplots illustrate the expression of three representative proteins associated with mitochondrial function and ISG15 signaling, showing consistent regulation across studies. Expression values correspond to log_2_ LFQ intensities obtained in our proteomic analyses of mock-infected and SARS-CoV-2–infected LC-HK2 cells at 48 and 96 hpi. All three proteins exhibited significant differences between conditions (*p* < 0.05, according to the Limma model). Each point represents an individual replicate measurement. (**E**) Heatmaps display the fold changes (FC) of representative mitochondrial proteins associated with oxidative phosphorylation, mitochondrial fission, and fusion, across the different datasets. The color scale represents unscaled fold-change values for clarity, and gray cells indicate missing data for a given protein in a particular study. The total proteome identified in each study was considered for this analysis, except for the dataset from Grossegesse et al. (2022) [60], which only reported differentially abundant proteins. For proteins related to mitochondrial fusion, some studies were omitted because no FC values were available for the selected genes.

To situate our work within the existing literature, our proteomic datasets were compared with previously published and publicly available datasets from studies employing lung-derived cells infected with SARS-CoV-2: Crozier et al., 2022; Babačić et al., 2023; Puray-Chavez et al., 2021; Stukalov et al., 2021; Grossegesse et al., 2022; Hatton et al., 2021 [56,57,58,59,60,61]. This integrative analysis revealed both shared and unique differentially abundant proteins between studies when comparing mock and infected conditions, as illustrated by the UpSet plot in Figure 5A. Enrichment analysis did not reveal mitochondrial-related processes in SARS-CoV-2–infected pulmonary cell models, as shown in Figure 5B, despite convergent transcriptomic signatures reported in multiple studies [63,64] and observations from individual proteomic reports [59,60]. This could be due to differences in experimental design across studies, as detailed in Supplementary Table S1, including cell lineage, multiplicity of infection, sampling time points post-infection, and proteomic acquisition methods (e.g., isobaric labeling vs. DIA), which markedly affect proteome coverage, missing values, and statistical power. Even with these limitations, we were able to identify a core set of 85 proteins that were differentially modulated in at least four independent studies.

These proteins were primarily associated with antiviral mechanisms, ISG15 conjugation, and interferon signaling, as shown in the enrichment analysis in Figure 5B. Among the proteins within this core set, several converge on MAVS-dependent antiviral signaling. These included *IFIT3*, which interacts with the N-terminal region of *TBK1* and bridges *TBK1* to *MAVS* at the mitochondria [65]; *IFIH1*, which upon ligand binding associates with *MAVS*/*IPS1* to initiate downstream antiviral response; and the *NFX1*-type zinc finger–containing protein 1, a dsRNA sensor that recognizes viral RNA and signals through MAVS [66]. In addition to these MAVS-associated factors, we identified glutathione peroxidase 1, a key regulator of mitochondrial redox homeostasis [67]; clusterin, which suppresses stress-induced apoptosis by stabilizing mitochondrial membrane integrity [68]; the succinate dehydrogenase [ubiquinone] iron–sulfur subunit; and mitochondrial carrier homolog 1. The complete list of proteins identified as differentially modulated in at least four studies is provided in Supplementary Table S7.

Next, we focused on proteins shared between our dataset and others and identified several related to mitochondrial dynamics, oxidative stress, and antiviral signaling. Overlapping proteins included MTCH2, GPX1, ISG15, STAT1, NFKB2, ARPC5, SMAD3, CETN2, RAB1B, CCN1, and P4HA2, representing key nodes connecting mitochondrial remodeling, interferon pathways, and cytoskeletal regulation, as shown in the correlation network in Figure 5C. Among these, MTCH2—a consistently detected protein—has been shown to act as a regulator of mitochondrial fission-fusion balance [69], while ISG15 and STAT1 indicate sustained activation of interferon-stimulated pathways [26], as illustrated in Figure 5D. Changes in GPX1 expression (Figure 5D) highlight the role of redox and oxidative homeostasis [70], whereas ARPC5 links mitochondrial positioning to actin cytoskeletal dynamics [71].

Comparison of mitochondrial-associated proteins across datasets revealed substantial variability not only in fold-change magnitude but also in detection consistency. As shown in Figure 5E, many metabolic enzymes and fission/fusion regulators were not uniformly quantified across studies, indicated by the grey regions in the heatmaps representing missing values. Even among proteins reliably detected, fold changes were generally modest and frequently divergent across datasets, with factors such as MFN1, MFN2, and DRP1 (DNM1L) showing opposing trends depending on the study and period of infection. This variability suggests that SARS-CoV-2–driven mitochondrial remodeling may not be fully captured by total protein abundance alone and likely reflects additional regulatory processes affecting protein turnover, subcellular localization, and post-translational modification states.

Together, our proteomic analysis and cross-study comparison revealed a consistent enrichment of antiviral and interferon-related pathways, particularly involving ISG15, STAT1, IFI35, and MAVS-associated signaling proteins, whereas mitochondrial-associated proteins showed more variable, modest, and dataset-dependent changes in total abundance. The modest and heterogeneous changes in total mitochondrial protein abundance prompted us to further investigate mitochondrial remodeling using targeted transcriptional and imaging approaches.

### 3.3. Proteomic Signatures of SARS-CoV-2 Infection Converge with Mitochondrial Morphology and Transcriptional Remodeling of the Mitochondrial Gene Program

**Figure 6:**
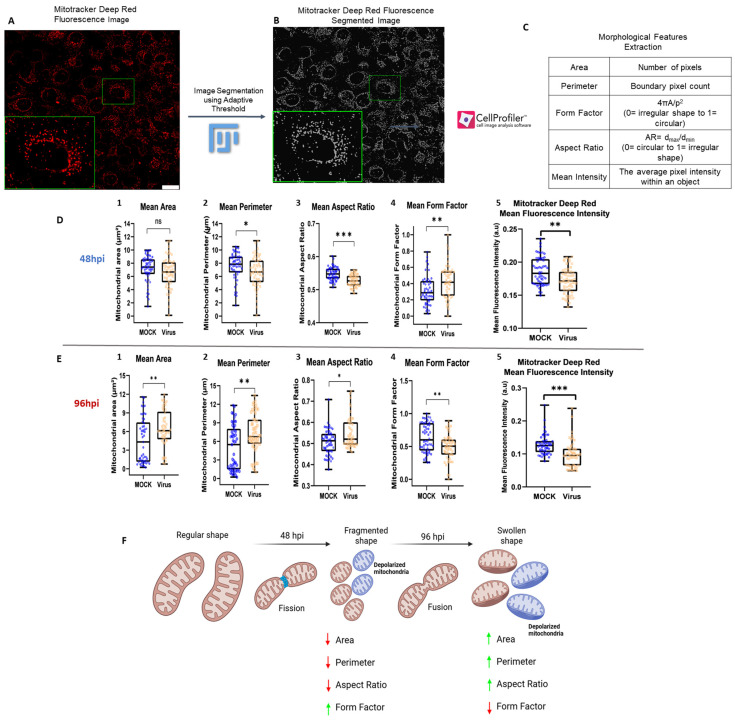
Mitochondrial morphology profiling following SARS-CoV-2 infection. (**A**) Representative confocal microscopy images of mitochondria stained with MitoTracker™ Deep Red in mock and SARS-CoV-2–infected cells at 48 hpi and 96 hpi. (**B**) Mitochondrial segmentation masks generated from panel (**A**) using the Adaptive Threshold plugin in FIJI/ImageJ (Fiji distribution of ImageJ, based on ImageJ2, including ImageJ 1.54s) illustrating binarization and object definition. The grey arrow indicates the image-processing workflow used to generate the mitochondrial segmentation mask from MitoTracker fluorescence images using FIJI/ImageJ. (**C**) Set of mitochondrial morphometric features extracted with CellProfiler, including area, perimeter, aspect ratio, form factor, and mean fluorescence intensity (as indicated in the panel). (**D**,**E**) Comparison between mock and SARS-CoV-2 for the extracted morphometric features at 48 hpi (**D**) and 96 hpi (**E**). Plots are shown as boxplots with individual data points, where each point represents one quantified measurement according to the analysis unit used in the pipeline (mitochondrial object/cell/field, as applicable). (**F**) Illustrative schematic summarizing the mitochondrial morphological profile at 48 hpi and 96 hpi. Statistical analyses were performed using an unpaired *t*-test or the Mann–Whitney U test, as appropriate based on data distribution. Data are presented as mean ± SD, and differences were considered statistically significant at *p* < 0.05 (*p* < 0.05; * *p* < 0.01; ** *p* < 0.001; *** *p* < 0.0001; ns, not significant). Scale bar = 100 µm.

To investigate mitochondrial morphology, we performed confocal microscopy experiments. Mitochondrial morphology analysis in LC-HK2 cells at 48 hpi demonstrated reduced perimeter, decreased aspect ratio, and increased form factor, indicating smaller and more rounded mitochondria consistent with fission (Figure 6). At 96 hpi, mitochondria displayed increased area and perimeter, together with higher aspect ratio and reduced form factor, reflecting elongated and irregular morphology compatible with fusion (Figure 6E,F). The transition from a fragmented phenotype at 48 hpi to elongated/perinuclear mitochondria at 96 hpi suggested that mitochondrial remodeling occurs dynamically during infection rather than as a static endpoint.

**Figure 7:**
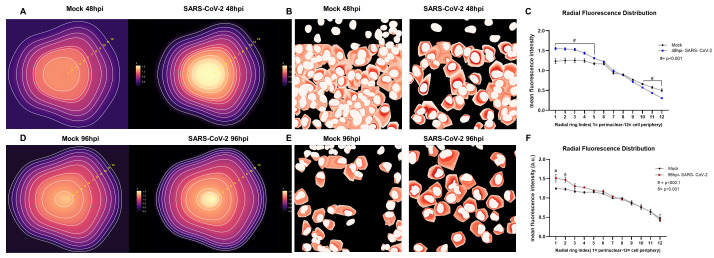
Topographic and quantitative analysis of mitochondrial distribution in mock and SARS-CoV-2–infected cells at 48 and 96 hpi. (**A**) Topographic maps of mitochondrial fluorescence intensity in mock and SARS-CoV-2–infected cells at 48 hpi, showing the radial distribution of MitoTracker™ DeepRed signal from the nuclear region toward the cell periphery across 12 concentric analytical rings. The maps were generated in R based on the mean fluorescence intensity profile obtained for each ring. (**B**) Heatmap of the radial distribution of mean fluorescence intensity per ring. At 48 hpi, the heatmap summarizes the relative contribution of each radial compartment (rings 1–12) for mock and SARS-CoV-2 conditions, highlighting changes in mitochondrial positioning along the nuclear–peripheral axis. (**C**) Line-plot representation of the mean fluorescence intensity per ring at 48 hpi, comparing mock and SARS-CoV-2–infected cells. (**D**–**F**) Corresponding analyses performed at 96 h post-infection (96 hpi): (**D**) topographic maps, (**E**) heatmap of the radial distribution of mean fluorescence intensity per ring, and (**F**) line plots of the mean fluorescence intensity per ring for mock and SARS-CoV-2. For all panels, data represent the mean of three independent experiments, each performed in technical triplicate. Statistical analyses were carried out in GraphPad Prism 10 using two-way ANOVA, followed by Bonferroni’s multiple comparisons test.

Furthermore, we evaluated the subcellular distribution of mitochondria in LC-HK2 cells. Confocal fluorescence intensity analyses of mitochondria labeled with MitoTracker™ revealed a perinuclear accumulation pattern in SARS-CoV-2–infected LC-HK2 cells at both 48 hpi and 96 hpi. This redistribution indicates a shift of mitochondria from a dispersed cytoplasmic arrangement toward a more centralized localization around the nucleus (Figure 7).

**Figure 8:**
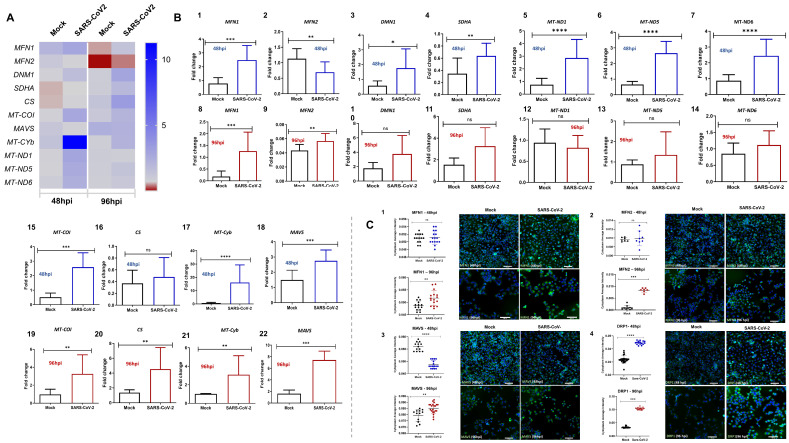
SARS-CoV-2 modulates mitochondrial dynamics and antiviral signaling at the mRNA and protein levels. (**A**) Heatmap summarizing the expression pattern of the indicated genes—*MFN1*, *MFN2*, *DNM1*, *SDHA*, *MT-ND1*, *MT-ND5*, *MT-ND6*, *MT-COI*, *CS*, *MT-Cyb*, and *MAVS* in mock and SARS-CoV-2–infected LC-HK2 cells at 48 hpi and 96 hpi. (**B1**–**22**) Gene expression analysis (fold change) of mitochondrial dynamics/respiratory genes *MFN1*, *MFN2*, *DNM1*, *SDHA*, *MT-ND1*, *MT-ND5*, *MT-ND6*, *MT-COI*, *CS*, *MT-Cyb*, and *MAVS* at 48 hpi (blue) and 96 hpi (red). (**C**) Confocal immunofluorescence microscopy showing MFN1 (**C1**), MFN2 (**C2**), MAVS (**C3**), and DRP1 (**C4**) (green) in mock and SARS-CoV-2–infected LCHK-2 cells at 48 hpi and 96 hpi, with nuclei counterstained with Hoechst 33342 (blue). Scatter plots report the quantitative image analysis from confocal micrographs, using cytoplasm average intensity for MFN1/MFN2/MAVS and DRP1; each dot represents one quantified measurement, and representative images are shown for each condition/time point (Scale bars correspond to 100 µm.). Gene-expression data are presented as the mean of three independent experiments, each performed in technical triplicate. Statistical analyses were carried out in GraphPad Prism 10 (GraphPad Prism Software). Pairwise comparisons between Mock- and SARS-CoV-2–infected cells were performed using the Mann–Whitney U test. Differences were considered statistically significant at *p* < 0.05. Significance levels are indicated as follows: * *p* < 0.05, ** *p* < 0.01, *** *p* < 0.001 and **** *p* < 0.0001; ns, not significant.

Consistent with this temporal morphological shift, targeted transcriptional analysis further supported the occurrence of altered mitochondrial dynamics during SARS-CoV-2 infection. Although mitochondrial proteins showed only subtle changes in the proteomic analysis, genes associated with mitochondrial fission/fusion, respiratory function, and mitochondrial content, including *MFN2*, *DRP1*/*DNM1L*, *COX*/*MT-COI* subunits, and citrate synthase (*CS*), showed significant transcriptional modulation (Figure 8).

**Figure 9:**
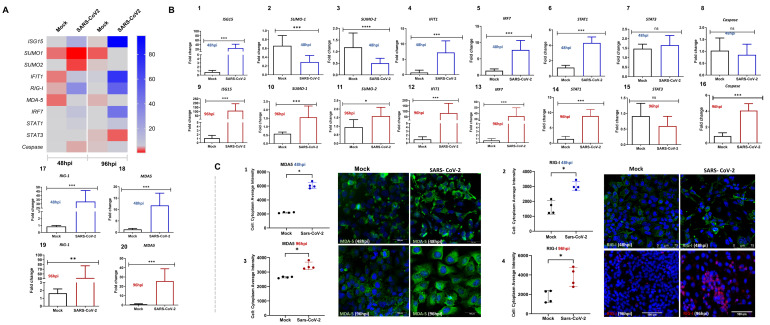
SARS-CoV-2 infection induces antiviral/IFN-related transcripts. (**A**) Heatmap summarizing the expression profile of *ISG15*, *SUMO1*, *SUMO2*, *IFIT1*, *IRF7*, *STAT1*, *STAT3*, Caspase, *RIG-I,* and *MDA-5*, in mock and SARS-CoV-2–infected LC-HK2 cells at 48 h post-infection (48 hpi) and 96 hpi. (**B1**–**20**) RT-qPCR fold-change analysis of *ISG15*, *SUMO1*, *SUMO2*, *IFIT1*, *IRF7*, *STAT1*, *STAT3*, Caspase, *RIG-I,* and *MDA-5* at 48 hpi (blue) and 96 hpi (red) comparing Mock vs. SARS-CoV-2. (**C1**–**4**) Representative confocal immunofluorescence images of MDA-5 (green) with nuclei counterstained with Hoechst 33342 (blue) and corresponding quantification of cytoplasmic fluorescence intensity at 48 hpi and 96 hpi. Representative confocal immunofluorescence images of RIG-I with the fluorescence channel shown as indicated, and nuclei counterstained with Hoechst 33342 (blue). The corresponding quantification shows cytoplasmic fluorescence intensity at 48 hpi (green) and 96 hpi (red). Each dot represents one quantified measurement from confocal microscopy (analysis unit as defined in the imaging pipeline). Scale bars: 100 µm. Gene-expression data are presented as the mean of three independent experiments, each performed in technical triplicate. Statistical analyses were carried out in GraphPad Prism 10 (GraphPad Software). Pairwise comparisons between mock- and SARS-CoV-2–infected cells were performed using the Mann–Whitney U test, and differences were considered statistically significant at *p* < 0.05. Significance levels are indicated as follows: * *p* < 0.05, ** *p* < 0.01, *** *p* < 0.001 and **** *p* < 0.0001; ns, not significant.

Gene expression analysis supported these findings, with upregulation of *DRP1* and downregulation of *MFN2*, together with increased *MT-CO1* and *MT-Cyb*, suggestive of compensatory mitochondrial transcription (Figure 8B). Concurrently, *MAVS*, *IRF7*, *ISG15*, and *IFIT1* were upregulated, indicating modulation of the *RIG-I*/*MAVS*–*IRF3*/*7–ISG* axis and an ongoing antiviral response (Figure 8C and Figure 9C). At the transcriptional level, additional modulation of mitochondrial complex I genes was observed. *MT-ND1*, *MT-ND5*, and *MT-ND6* at 48 hpi were upregulated (Figure 8B(5–7)) and 96 hpi while their expression returned to basal levels (Figure 8B(12–14)).

These changes correlated with increased *MFN2* and *CS*, along with sustained *MT-CO1/MT-Cyb* expression, consistent with mitochondrial biogenesis. Antiviral genes (*MAVS*, *IRF7*, *ISG15*, and *IFIT1*) remained upregulated, while *SUMO1/2* expression was reduced, suggesting altered post-translational regulation (Figure 8 and Figure 9). Early reduction of *SUMO1* and *SUMO2* may contribute to weakening innate immune signaling through disruption of RIG-I and *MDA5* regulatory pathways, whereas increased *SUMO1/2* expression at later stages of infection suggests that SARS-CoV-2 may exploit SUMOylation to enhance viral protein function, promote immune evasion, and facilitate nucleocapsid protein nuclear translocation [72,73]. The transcriptional modulation of mitochondrial-related genes revealed a biphasic pattern of regulation, with early fission-associated signatures followed by late fusion and biogenesis responses (Table 1). Given that mitochondrial dynamics and antiviral signaling are regulated by post-translational modifications, we evaluated SUMO2/3 to determine whether changes in this post-translational regulatory pathway occurred concomitantly with mitochondrial morphological remodeling and antiviral signaling during SARS-CoV-2 infection.

### 3.4. SUMO2/3 Modulation Correlates with Mitochondrial Remodeling During SARS-CoV-2 Infection in LC-HK2 Cells

**Figure 10:**
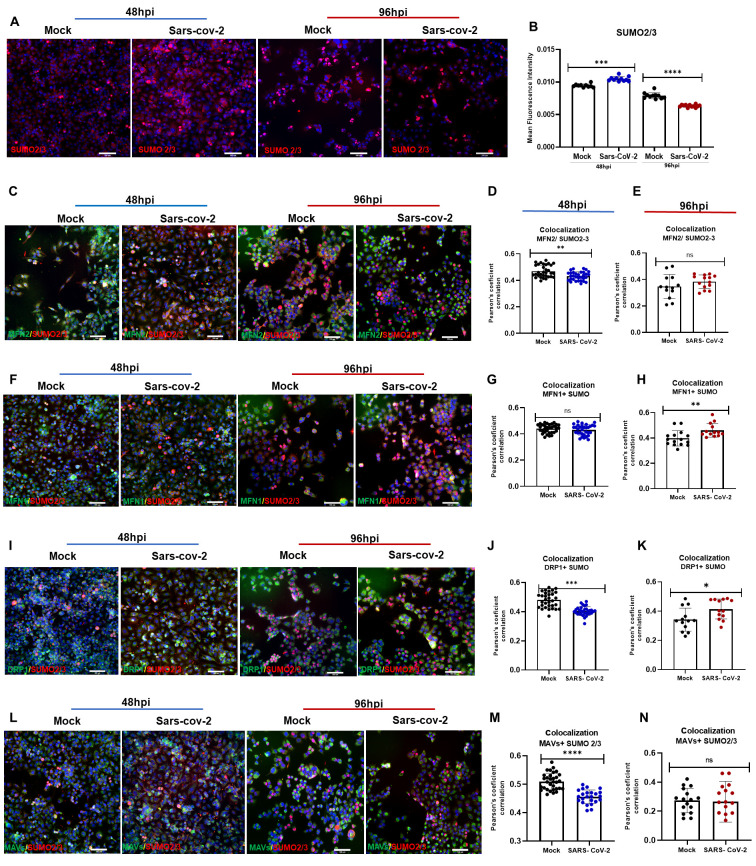
SARS-CoV-2 modulates SUMO2/3 abundance and its colocalization with mitochondrial dynamics and antiviral signaling proteins. (**A**) Representative HCS images showing SUMO2/3 in mock and SARS-CoV-2–infected cells at 48 hpi and 96 hpi (SUMO2/3, red; nuclei, Hoechst 33342/blue). (**B**) Quantification of SUMO2/3 mean fluorescence intensity, showing increased signal at 48 hpi and reduced signal at 96 hpi in infected cells compared with mock. (**C**,**F**,**I**,**L**) Representative merged images of SUMO2/3 with MFN2, MFN1, DRP1, and MAVS, respectively, in mock and infected cells at 48 hpi and 96 hpi. (**D**,**E**,**G**,**H**,**J**,**K**,**M**,**N**) Colocalization quantification for each protein with SUMO2/3 at 48 hpi and 96 hpi, expressed as Pearson’s correlation coefficient. Representative images were obtained from HCS acquisitions, whereas quantification and colocalization analyses were performed using confocal images for higher precision. Each dot represents one quantified measurement (analysis unit defined in the imaging pipeline). Statistical analyses were performed using an unpaired *t*-test or the Mann–Whitney U test, as appropriate based on data distribution. Data are presented as mean ± SD, and differences were considered statistically significant at *p* < 0.05 (* *p* < 0.05; ** *p* < 0.01; *** *p* < 0.001; **** *p* < 0.0001; ns, not significant). Scale bar = 100 µm.

First, we evaluated whether SARS-CoV-2 infection was associated with changes in SUMO2/3 abundance and subsequently examined its spatial association with mitochondria. Confocal microscopy analyses revealed dynamic modulation of SUMO2/3 expression in LC-HK2 cells infected with SARS-CoV-2 at 48 and 96 h post-infection (hpi). At 48 hpi, SUMO2/3 intensity was markedly increased relative to mock-infected controls, whereas a significant reduction was observed at 96 hpi (Figure 10A,B). Colocalization analysis between SUMO2/3 and mitochondrial proteins or antiviral signaling proteins showed distinct temporal profiles showed distinct temporal profiles. At 48 hpi, colocalization with MFN2, DRP1, and MAVS was decreased compared with mock-infected cells (Figure 10C,D,I,J,L,M). Consistent with altered SUMO2/3-associated regulation of mitochondrial dynamics and antiviral signaling, MFN1 remained unchanged at this time point. By 96 hpi, colocalization of MFN1 and DRP1 with SUMO2/3 was observed, whereas MFN2 and MAVS showed no significant differences compared with mock controls (Figure 10C,F–J,N).

Together, these findings indicate that SARS-CoV-2 infection is associated with temporally regulated modulation of SUMO2/3, characterized by increased SUMO2/3 signal at 48 hpi and reduced signal at 96 hpi, together with time-dependent changes in its colocalization with mitochondrial dynamics proteins and MAVS. Rather than demonstrating immune evasion directly, this pattern suggests that SUMO2/3 remodeling may contribute to changes in mitochondrial organization and to the regulation of the MAVS/RLR antiviral signaling axis during infection.

Given that SUMO2/3 remodeling occurred concomitantly with changes in mitochondrial organization and MAVS-associated antiviral signaling, we next examined whether another antiviral ubiquitin-like modifier, ISG15, followed a related spatial pattern in the same infection context. This analysis was further supported by the consistent modulation of ISG15 in the proteomic and transcriptional datasets and by previous evidence linking ISG15 to mitochondrial homeostasis. Therefore, we investigated the spatial relationship between ISG15 and mitochondrial fusion markers.

### 3.5. Functional Profiling of ISG15 Expression

**Figure 11:**
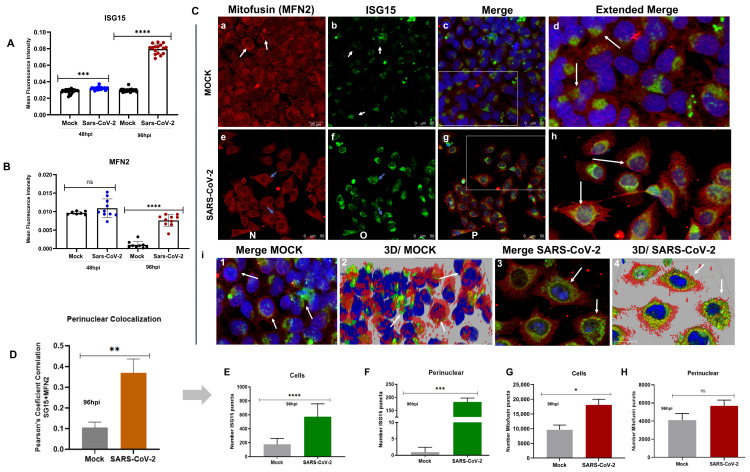
Expression profiles and spatial organization of ISG15 and MFN2 in mock and SARS-CoV-2–infected LC-HK2 cells. (**A**,**B**) Mean fluorescence intensity of ISG15 (**A**) and MFN2 (**B**) quantified from confocal immunofluorescence images at 48 hpi and 96 hpi, comparing mock and SARS-CoV-2 conditions. (**C**) Representative confocal images showing MFN2 (red) and ISG15 (green) distribution and their merged signal, including magnified views; 3D confocal reconstructions illustrate ISG15/MFN2 spatial overlap near the nucleus in infected cells. In panel C, subpanels (**a**–**d**) correspond to mock cells: (**a**) Mitofusin 2 (MFN2, red), (**b**) ISG15 (green), (**c**) merged image showing MFN2/ISG15/nuclear staining, and (**d**) enlarged merged image. Subpanels (**e**–**h**) corresponds to SARS-CoV-2-infected cells: (**e**) MFN2, (**f**) ISG15, (**g**) merged image showing MFN2/ISG15/nuclear staining, and (**h**) enlarged merged image. Images (**1**–**4**) represent selected merged images and 3D reconstructions: (**1**) merged mock image, (**2**) 3D reconstruction of mock cells, (**3**) merged SARS-CoV-2 image, and (**4**) 3D reconstruction of SARS-CoV-2-infected cells. (**D**) Perinuclear colocalization between ISG15 and MFN2 at 96 hpi, expressed as Pearson’s correlation coefficient. (**E**–**H**) Puncta quantification at 96 hpi assessing spatial distribution: number of ISG15 puncta per cell (**E**) and in the perinuclear region (**F**), and number of MFN2 puncta per cell (**G**) and in the perinuclear region (**H**), comparing mock and SARS-CoV-2–infected cells. Each dot represents one quantified cell (analysis unit defined in the image pipeline). Green: ISG15 (donkey FITC-conjugated anti-rabbit IgG); red: MFN2 (Alexa Fluor™ 647–conjugated anti-mouse); blue: nuclei (Hoechst 33342). Scale bars as indicated. Statistical comparisons between mock and SARS-CoV-2 were performed using the Mann–Whitney U test (nonparametric). Values of *p* < 0.05 were considered statistically significant. Significance levels are indicated as follows: * *p* < 0.05, ** *p* < 0.01, *** *p* < 0.001, and **** *p* < 0.0001; ns, not significant.

To investigate the spatial distribution of ISG15 in relation to mitochondrial fusion-associated remodeling, we performed immunolabeling for ISG15 and MFN2. MFN2 was selected based on the mitochondrial morphology data showing an elongated/perinuclear mitochondrial phenotype at 96 hpi, compatible with fusion-associated remodeling. Confocal microscopy analysis showed that ISG15 fluorescence intensity was increased in SARS-CoV-2–infected LC-HK2 cells, with an asymmetric cytoplasmic distribution and prominent perinuclear accumulation at 96 hpi (Figure 11A,C(h)). In contrast, mock-treated cells displayed lower and more diffuse ISG15 signal, with minimal spatial overlap with MFN2 (Figure 11C(d)).

MFN2 fluorescence intensity was also increased in infected cells at 96 hpi (Figure 11B), although its overall distribution pattern was not markedly different between mock and infected cells (Figure 11E). Notably, in SARS-CoV-2–infected cells, ISG15 showed partial colocalization with MFN2-positive perinuclear mitochondrial regions, as supported by Pearson’s correlation coefficient values above 0.4 (Figure 11D). These findings indicate a partial spatial association between ISG15 and MFN2-positive mitochondrial regions during the late phase of infection. However, this association should not be interpreted as evidence that ISG15 directly drives mitochondrial fusion or hyperfusion. Rather, the observed phenotype may reflect a coordinated cellular rearrangement involving mitochondrial remodeling, antiviral/stress-related ISG15 responses, and organelle quality-control pathways during SARS-CoV-2 infection.

Finally, to determine whether these intracellular mitochondrial and antiviral alterations were associated with an extracellular inflammatory output, we analyzed cytokines and secreted ISG15 in conditioned media.

### 3.6. Secreted ISG15 and Pro-Inflammatory Cytokine Profile in SARS-CoV-2–Infected LC-HK2 Cells

**Figure 12:**
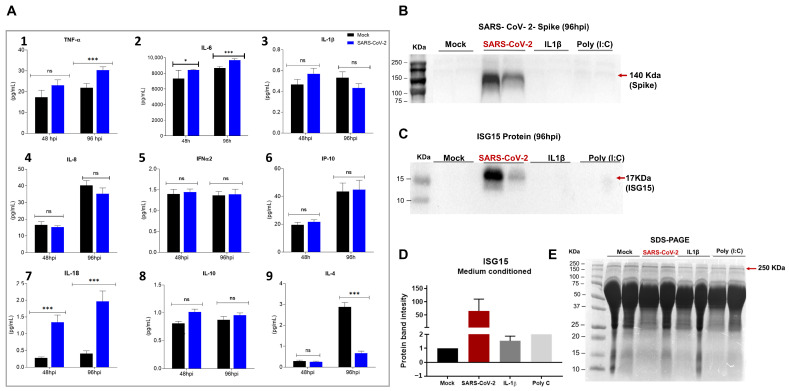
Secreted ISG15 and SARS-CoV-2 S protein in conditioned medium and cytokine release profile in LC-HK2 cells. (**A**) Multiplex cytokine quantification in cell-free supernatants collected at 48 hpi and 96 hpi using the Milliplex^®^ MAP Human Cytokine/Chemokine Magnetic Bead Panel (HCYTOMAG-60K and HSTCMAG-28SK; EMD Millipore). Subpanels (1–9) correspond to the following analytes: (1) TNF-α, (2) IL-6, (3) IL-1β, (4) IL-8, (5) IFNα2, (6) IP-10, (7) IL-18, (8) IL-10, and (9) IL-4. (**B**) The SARS-CoV-2 spike (S) protein (~140 kDa) was detected only in CM from infected cells. (**C**,**D**) Elevated levels of ISG15 (17 kDa) were detected in the conditioned medium (CM) of SARS-CoV-2–infected LC-HK2 cells compared with mock cells. (**E**) SDS-PAGE of cell-free supernatants from LC-HK2 cells under SARS-CoV-2, mock, Poly(I:C), and IL-1β conditions. For Western blot and SDS-PAGE analysis of conditioned medium, samples from biological triplicates belonging to the same experimental condition were pooled before concentration due to the limited sample volume. Each of the two lanes shown for each condition represents an independent concentrated pool derived from biological triplicates, and not a technical duplicate of the same pooled sample. IL-1β and Poly(I:C) lanes correspond to internal cytokine/ISG15 release–inducing controls, not replicates of the SARS-CoV-2–infected condition. A band at ~250 kDa in the Coomassie-stained gel was used as a normalization reference, and ISG15 and S protein signals were normalized to the total protein profile obtained by Coomassie staining, as previously described. This ~250 kDa band was used only as an internal intra-gel reference for semi-quantitative normalization and was not considered a housekeeping protein or a universal representation of the total secreted proteome. Data were acquired on a Luminex™ 200 system using xPONENT 4.3 and analyzed with Milliplex^®^ Analyst 5.1; cytokine concentrations are reported in the pg/mL range. Analyses were performed with biological triplicates and experimental duplicates, and cytokine release–inducing treatments (IL-1β, 1 ng/mL; Poly(I:C), 10 μg/mL) were included as internal controls. SDS-PAGE, sodium dodecyl sulfate-polyacrylamide gel electrophoresis; SARS-CoV-2, severe acute respiratory syndrome coronavirus 2; S, spike; ISG15, interferon-stimulated gene 15; IL, interleukin; Poly(I:C), polyinosinic/polycytidylic acid. Values of *p* < 0.05 were considered statistically significant. Significance levels are indicated as follows: * *p* < 0.05, *** *p* < 0.001, ns, not significant.

To determine whether the mitochondrial and interferon-related alterations observed in SARS-CoV-2–infected LC-HK2 cells were associated with changes in the inflammatory profile, we analyzed cytokines and chemokines released into cell-free supernatants at 48 and 96 h post-infection (hpi) (Figure 12). This approach allowed the evaluation of the cell-intrinsic inflammatory response of lung epithelial cells in the absence of a complex multicellular inflammatory niche. Multiplex analysis revealed that SARS-CoV-2 infection elicited a selective pro-inflammatory response. Among the analytes examined, IL-6 and IL-18 (Figure 12A(2,7)) were consistently elevated in infected cultures at both 48 and 96 hpi compared with mock-treated controls, with a more pronounced increase at 96 hpi. At this later time point, TNF-α levels were also increased, whereas the anti-inflammatory cytokine IL-4 was reduced, indicating a shift toward a sustained pro-inflammatory state (Figure 12A(1,9)). In contrast, canonical antiviral or inflammasome-associated mediators, including IL-1β and IFN-α2, did not show a significant induction upon infection (Figure 12A(3,5)). In parallel, analysis of the same cell-free supernatants collected at 96 hpi demonstrated increased levels of secreted ISG15 in the conditioned medium (CM) of SARS-CoV-2–infected LC-HK2 cultures relative to mock controls (Figure 12C,D). The SARS-CoV-2 spike (S) protein (~140 kDa) was detected exclusively in CM from infected cells, confirming viral infection. ISG15 and S protein signals were normalized to the total protein profile obtained by Coomassie staining of SDS-PAGE gels from cell-free supernatants, using the ~250 kDa band as a normalization reference, as previously described.

Coomassie-stained SDS-PAGE of cell-free conditioned medium samples was used to assess the protein profile and comparable loading among experimental conditions. The prominent approximately 250 kDa band, consistently detected and non-saturated across lanes, was used as an internal intra-gel reference for semi-quantitative normalization of ISG15 and SARS-CoV-2 S protein signals. This band was not considered a housekeeping protein or a universal representation of the total secreted proteome, but rather a technical reference to support relative comparison between supernatant samples processed in parallel.

## 4. Discussion

Despite the successful development and widespread deployment of vaccines against COVID-19, the continuous emergence of new SARS-CoV-2 variants still poses a major threat to global health and may erode the progress achieved in limiting viral transmission. Understanding the molecular mechanisms that govern the interaction between SARS-CoV-2 and host cells, therefore, remains a central scientific challenge, requiring complementary experimental and computational approaches [87,88].

In this context, we demonstrate that lung-derived cells (LC-HK2) are highly susceptible to SARS-CoV-2 infection. This model proved suitable for dissecting virus–host interactions in human lung cells, enabling investigation of the biological effects of SARS-CoV-2 proteins, modulation of antiviral responses, and the cellular remodeling that accompanies infection. Importantly, LC-HK2 cells have also been successfully employed in antiviral drug screening approaches, as they recapitulate key features of the human pulmonary environment relevant to viral entry and replication. Previous studies using this model have shown that LC-HK2 cells reliably discriminate the antiviral efficacy of host-targeting compounds, such as cathepsin inhibitors, and allow assessment of cytopathic effects, viral replication, and host cell viability in a physiologically relevant context [45].

Together, these attributes support the use of LC-HK2 cells as a complementary lung-derived platform not only for mechanistic studies of SARS-CoV-2 pathogenesis, but also for the evaluation of candidate therapeutics aimed at modulating virus–host interactions in lung epithelial cells. Although LC-HK2 cells are a transformed tumor-derived cell line and, therefore, do not fully recapitulate the complexity of primary airway epithelial cells or the in vivo pulmonary microenvironment, they provide a reproducible model for investigating SARS-CoV-2–associated mitochondrial remodeling and antiviral-related cellular responses. In the present study, this model was particularly useful for integrating mitochondrial morphology, antiviral transcriptional responses, ISG15-associated signaling, SUMO2/3 modulation, and cytokine output within the same infection context. This integrated approach allowed us to evaluate whether these events occur as part of a coordinated mitochondrial–immune remodeling program rather than as isolated infection-associated observations.

### 4.1. Viral Entry and Receptor Regulation

Alterations in viral entry receptor expression were evident in SARS-CoV-2–infected LC-HK2 cells, particularly involving *ACE2*. A marked reduction in *ACE2* protein levels was observed at 96 hpi, consistent with previous reports showing that both SARS-CoV and SARS-CoV-2 promote *ACE2* downregulation through receptor internalization following viral entry [89,90]. This finding supports the occurrence of productive virus–host interaction in LC-HK2 cells and provides an initial context for interpreting the subsequent mitochondrial and antiviral remodeling observed during infection.

### 4.2. Proteomic Evidence of Temporally Regulated Antiviral and Mitochondrial Remodeling During SARS-CoV-2 Infection

The proteomic analyses performed in LC-HK2 cells, together with comparisons across publicly available lung-derived SARS-CoV-2 datasets, provide an initial framework to discuss how infection is associated with a coordinated but heterogeneous remodeling of antiviral signaling, mitochondrial-associated pathways, and post-translational regulatory processes over time. The global and comparative proteomic analysis shows that SARS-CoV-2 infection in LC-HK2 cells induces modulation of host pathways, with a consistent enrichment of antiviral and ISG15-related signatures, but only modest and scattered changes in classical mitochondrial markers. The presence of multiple viral proteins at both 48 and 96 hpi, together with enrichment of terms related to viral process, chromatin remodeling, and mitochondrial gene expression among proteins exclusive to infected cells, confirms that LC-HK2 cells support productive infection and sustain virus–host interactions over time. The temporal pattern of differentially abundant proteins suggests a shift from early metabolic and structural remodeling toward more pronounced immune and stress responses at later stages of infection. Downregulation of *CHCHD2* and *FIS1* at 48 hpi, followed by modulation of *ATP5IF1*, *NDUFS5*, *MTCH2*, and *DIABLO-2* at 96 hpi, indicates that mitochondrial dynamics, electron transport chain function, and apoptosis-related pathways are selectively targeted during SARS-CoV-2 infection. In parallel, the sustained upregulation of ISG15, STAT1, and IFI35 across both time points suggests persistent antiviral and ISG-associated signaling, despite the presence of viral antagonism. Thus, the proteomic data suggest that mitochondrial involvement during infection may not be reflected exclusively by large changes in total abundance of canonical mitochondrial proteins, but also by coordinated changes in stress, antiviral, redox, cytoskeletal, and post-translational regulatory pathways. This pattern is consistent with previous reports linking SARS-CoV-2 infection to chronic interferon stimulation, altered immunometabolism, and mitochondrial dysfunction in lung models and patient samples [15,63,64].

When our datasets were integrated with other proteomic studies in lung-derived systems, only a limited overlap of mitochondrial-related proteins was observed, despite convergent evidence of mitochondrial involvement in transcriptomic and functional studies [15,63,64]. This restricted convergence likely reflects differences in experimental design—including cell type, viral strain, multiplicity of infection, sampling time, and quantification strategy—as well as intrinsic technical constraints of discovery proteomics. For instance, the use of isobaric labeling and data-independent acquisition in some studies may reduce missing values and enhance quantification depth compared with data-dependent label-free approaches. Nevertheless, the core set of overlapping proteins we identified—such as MTCH2, GPX1, ISG15, STAT1, NFKB2, SMAD3, ARPC5, and RAB1B—highlights functional intersections between mitochondrial remodeling, oxidative stress, cytoskeletal organization, and interferon-associated signaling [26,69,70]. The heterogeneity of fold changes for mitochondrial markers across datasets, as seen in the heatmaps, suggests that post-translational modifications, protein relocalization, and organelle-specific remodeling may be more critical than large changes in total protein abundance for mitochondrial reprogramming during SARS-CoV-2 infection. Mechanisms such as SUMOylation, ISGylation, and protease-mediated cleavage have been implicated in fine-tuning mitochondrial quality control and antiviral responses [24,91,92]. Previous reports have shown that viral proteins, including PLpro, ORF9b, and Nsp1, can interfere with these pathways by altering the subcellular localization and modification status of key host factors, as well as dampening MAVS-dependent signaling [63,93,94]. Experimental and computational work further suggests that N, M, ORF10, and ORF9b can modulate mitochondrial protein expression and localization via post-translational mechanisms [95,96,97,98].

In this context, our data support the interpretation that SARS-CoV-2 infection is associated with immunometabolic remodeling in LC-HK2 cells, in which mitochondrial stress, antiviral/ISG-related signaling, and post-translational regulation converge. However, whether these changes directly sustain viral persistence or primarily reflect host stress adaptation requires further functional investigation.

### 4.3. SARS-CoV-2 Infection Promotes Temporal Remodeling of Mitochondrial Dynamics, ISG15–Mitochondrial Spatial Association, and Imbalanced Antiviral Signaling in LC-HK2 Cells

Together, our data support a temporal model in which SARS-CoV-2 infection induces a biphasic mitochondrial–antiviral remodeling program in LC-HK2 cells. At 48 hpi, infected cells displayed morphological and transcriptional features consistent with mitochondrial stress and fission, including increased DRP1 expression, reduced MFN2 expression, and transient upregulation of mitochondrial-encoded respiratory genes. This early response occurred in parallel with the induction of RIG-I/MDA5-related antiviral transcripts, MAVS, IRF7, ISG15, STAT1, and IFIT1, indicating regulation of an antiviral transcriptional program. However, this transcriptional regulation was not accompanied by a proportional increase in IFN-α2 secretion, suggesting that SARS-CoV-2 infection establishes an antiviral-alert state with incomplete downstream interferon output.

Our findings indicate that SARS-CoV-2 infection induces a dynamic remodeling of mitochondrial organization in LC-HK2, transitioning from an early fragmented phenotype toward a state characterized by partial recovery of mitochondrial fusion and biogenesis pathways at later stages of infection. This remodeling is evidenced by the perinuclear accumulation of mitochondria at both 48 and 96 hpi and by the emergence of hyperfused mitochondrial structures at 96 hpi, a phenotype frequently associated with cellular stress responses, antiviral signaling platforms, and impaired mitochondrial quality control [90,99]. A central observation of this study is the spatial convergence of ISG15 and MFN2 within the perinuclear mitochondrial network at 96 hpi. The increased abundance and asymmetric perinuclear distribution of ISG15, together with its partial colocalization with MFN2, suggest an association between innate immune signaling and mitochondrial dynamics during SARS-CoV-2 infection. Importantly, this colocalization should be interpreted as a spatial association between ISG15-related antiviral/stress responses and mitochondrial remodeling, rather than as direct evidence that ISG15 drives mitochondrial fusion or hyperfusion. In this sense, previous works have shown that mitochondrial fusion mediated by MFN2 has been implicated in stabilizing mitochondrial networks under stress and in regulating MAVS-dependent antiviral signaling [100]. ISGylation of mitochondrial proteins has been shown to modulate mitophagy in vaccinia virus–infected cells [27], and perinuclear ISG15–MFN2 colocalization has been implicated in the regulation of mitophagy in neurological disease models [28]. This finding is consistent with reports that ISG15 regulates mitochondrial respiration, ROS production, and mitophagy, acting as a critical node in mitochondrial homeostasis during infection [16,30].

Studies have shown that the absence of ISG15 leads to the accumulation of elongated and dysfunctional mitochondria in the perinuclear region, suggesting that ISGylation contributes to the regulation of mitochondrial morphology and turnover [27]. Previous studies have also demonstrated that a significant fraction of free ISG15 localizes to mitochondria, where both ISG15 and ISGylated proteins are enriched in mitochondrial compartments, including the intermembrane space and the inner mitochondrial membrane [22,27,101]. Proteomic analyses further indicate that numerous mitochondrial proteins are potential targets of ISGylation, supporting the role of ISG15 in the regulation of mitochondrial function under conditions of cellular stress and viral infection [102]. Importantly, ISG15 has also been associated with pathways involved in mitochondrial fission and fusion, including the modulation of proteins such as DRP1 and components related to Parkin-mediated mitophagy. In this context, the perinuclear region appears to serve as an important site for mitochondrial quality control and immune-associated mitochondrial signaling [103]. At 96 hpi, the mitochondrial phenotype shifted toward elongated and perinuclear networks, accompanied by increased MFN2 and citrate synthase expression and sustained ISG15 abundance. The perinuclear colocalization of ISG15 with MFN2-positive mitochondrial structures suggests a spatial convergence between mitochondrial remodeling and ISG15-associated antiviral/stress responses. We interpret this association cautiously as evidence of coordinated mitochondrial–immune remodeling rather than direct proof of a mechanistic interaction. In this context, ISG15 may mark mitochondria-associated stress or quality-control compartments, whereas MFN2-associated mitochondrial fusion may reflect either a host attempt to preserve mitochondrial integrity or a virus-associated remodeling state that supports metabolic adaptation.

However, this interpretation remains hypothetical and requires targeted functional studies to determine whether ISG15 directly participates in mitochondrial quality control or whether its localization primarily reflects a stress-associated antiviral state.

In parallel, infected cells displayed increased levels of MDA5, RIG-I, and STAT1. MAVS also exhibited increased transcript expression at both 48 hpi and 96 hpi, despite a transient reduction in protein levels at 48 hpi. In addition, downstream antiviral-related genes were increased at later stages of infection, while IFN-I output remained comparatively modest in our system. Thus, our findings suggest a partial uncoupling between RLR/MAVS-related transcriptional regulation and downstream interferon production. This interpretation is consistent with the robust induction of ISG-related transcripts in parallel with limited IFN-α2 secretion.

Proteomic profiling further supported the presence of antiviral and ISG-associated signaling by identifying ISG15, STAT1, and IFI35 among the most strongly modulated host proteins. Notably, previous studies have shown that SARS-CoV-2 encodes the papain-like protease (PLpro), which exhibits deISGylase activity and has been shown to antagonize ISGylation, favoring immune evasion [104]. In our model, the increased intracellular levels of ISG15, together with reduced levels of mitochondrial antiviral components and limited induction of IFN-related cytokines, suggest altered regulation of ISG15-associated pathways during SARS-CoV-2 infection. These data support the presence of an ISG15-rich antiviral/stress state but do not establish whether ISG15 functions primarily as a protective host response, a marker of mitochondrial stress, or a pathway counteracted by viral proteins.

In parallel with these intracellular events, we detected elevated levels of extracellular ISG15 in supernatants from infected LC-HK2 cells at both 48 and 96 hpi. Secreted ISG15 retains biological activity and has been described to modulate interferon-stimulated gene responses in recipient cells, while also functioning as a cytokine-like mediator that enhances NK cell function and IFN-γ production [29,32]. While this extracellular signaling may initially contribute to antiviral defense, sustained ISG15 release in the context of mitochondrial dysfunction may also promote increased inflammatory signaling. Consistent with this notion, infected LC-HK2 cells displayed a selective pro-inflammatory cytokine profile characterized by increased IL-6, IL-18, and TNF-α, together with reduced IL-4, indicative of a shift toward a persistent pro-inflammatory state [30,105]. The coexistence of extracellular ISG15, selective pro-inflammatory cytokine release, and limited IFN-α2 secretion further supports the concept of an imbalanced antiviral-inflammatory response rather than a fully effective interferon response. Although DRP1 ISGylation has not yet been directly characterized in the context of SARS-CoV-2 infection, Das and Chakrabarti (2024) [24] demonstrated that ISG15 conjugation to DRP1 promotes mitochondrial fission, whereas deISGylation shifts the balance toward mitochondrial hyperfusion in stressed cells. In light of the robust ISG15 upregulation and the dynamic changes in mitochondrial morphology observed in SARS-CoV-2–infected LC-HK2 cells, it is possible that a similar ISG15–DRP1 regulatory axis may contribute to the transition from fragmented mitochondria at 48 hpi to hyperfused, perinuclear networks at 96 hpi. Nevertheless, because DRP1 ISGylation was not directly measured in our system, this interpretation should be considered a literature-supported hypothesis rather than a conclusion directly demonstrated by our data. Integrating this framework with the accumulating evidence that COVID-19 is linked to mitochondrial dysfunction and altered mitochondrial quality control further supports the notion that ISG15-dependent regulation of DRP1 and MFN2 may represent a potentially relevant, yet underexplored, component of SARS-CoV-2–driven mitochondrial remodeling. Additionally, evidence from MERS-CoV and SARS-CoV infections in bronchial epithelial cells indicates that chronic coronavirus infection reduces mitophagy despite its role in host defense [92,94,106,107,108].

### 4.4. Dynamic SUMO2/3 Modulation Accompanies Mitochondrial Remodeling and Altered MAVS/RLR Antiviral Signaling in SARS-CoV-2–Infected LC-HK2 Cells

Our findings indicate that SARS-CoV-2 infection induces a temporally regulated remodeling of mitochondrial dynamics coupled to dynamic modulation of the host SUMOylation machinery. At 48 hpi, LC-HK2 cells exhibited morphological and transcriptional signatures consistent with stress-induced mitochondrial fission, including increased *DRP1* expression and transient upregulation of Complex I genes (*MT-ND1*, *MT-ND5*, and *MT-ND6*). We hypothesize that this early response may reflect an acute mitochondrial stress adaptation aimed at preserving electron transport and ATP production under antiviral signaling pressure, even though functional assays are necessary to prove this hypothesis. Concomitantly, upregulation of the *MAVS*–*IRF*–*ISG* axis supports engagement of innate immune defenses. Together, these observations place SUMO2/3 modulation within a broader temporal response that includes mitochondrial stress, antiviral transcriptional activation, and incomplete downstream interferon output.

By 96 hpi, mitochondria shifted toward a fusion- and biogenesis-associated phenotype, as evidenced by increased MFN2 and citrate synthase expression and remodeling into elongated or irregular structures. It is possible that this transition may represent either a host attempt to restore bioenergetic homeostasis or a viral strategy to reprogram mitochondrial metabolism to sustain replication. The lack of sustained Complex I upregulation at 96 hpi suggests that early compensatory mechanisms are not maintained, potentially due to viral interference with mitochondrial bioenergetics. We observed early elevation of SUMO2/3 (48 hpi), followed by reduction at 96 hpi, indicating dynamic regulation of SUMOylation during infection. Temporal SUMO remodeling has been described in viral infections [109], supporting the interpretation that early SUMO2/3 accumulation reflects an acute stress-adaptive response. Notably, SARS-CoV-2 infection has been shown to trigger cytoplasmic relocalization of SUMO1 and perinuclear redistribution of SUMO2 [109], aligning with our observation of perinuclear mitochondrial reorganization. However, our data do not demonstrate that SUMO2/3 remodeling directly mediates immune evasion. Instead, they suggest that temporal SUMO2/3 modulation may contribute to changes in mitochondrial organization and to regulation of the MAVS/RLR antiviral signaling axis during SARS-CoV-2 infection.

Previous works suggest that, mechanistically, SARS-CoV-2 actively exploits the SUMO system. The viral nucleocapsid (N) protein undergoes SUMOylation, particularly at residue K65, a modification essential for oligomerization and nuclear translocation [73]. The host E3 ligase TRIM28 mediates SUMO conjugation to N, promoting viral liquid–liquid phase separation (LLPS) and suppression of innate immunity [110]. Additionally, the viral protease Nsp5 enhances MAVS SUMOylation, increasing MAVS protein stability but redirecting signaling toward NF-κB activation rather than type I interferon production [76]. This mechanism shifts the antiviral response toward a pro-inflammatory profile, contributing to increased cytokine production. These literature findings provide a mechanistic context for interpreting our SUMO2/3 observations, but our study should be understood as demonstrating temporal SUMO2/3 remodeling and altered colocalization patterns, not direct viral exploitation of SUMOylation.

Beyond immune signaling, it has been demonstrated in the literature that SUMOylation also regulates viral entry and structural stability. ACE2, the SARS-CoV-2 entry receptor, is modified by SUMO3; inhibition of this modification promotes receptor degradation via autophagy [111], indicating that viral entry itself depends on host SUMO machinery. Viral structural and non-structural proteins further interface with SUMO pathways: spike (S) and nucleocapsid (N) are SUMO-modified to regulate virion assembly and replication, while Nsp5 and Nsp14 manipulate host SUMO-related factors to stabilize viral replication complexes. Collectively, these studies support the broader concept that SARS-CoV-2 infection can affect SUMO-associated host pathways. In our model, the observed changes in SUMO2/3 abundance and colocalization suggest that this regulatory layer may be linked to mitochondrial remodeling and altered antiviral signaling, although causality remains to be established.

In our model, increased colocalization of MFN1 and DRP1 with SUMO2/3 at 96 hpi suggests that SUMOylation may participate in the regulation of the mitochondrial fission–fusion machinery. Previous seminal studies have established that depolarized mitochondria accumulate PINK1, recruit Parkin, and undergo ubiquitin-dependent clustering prior to mitophagic clearance [112,113]. These processes depend on p62/SQSTM1-mediated aggregation and microtubule-based transport toward the perinuclear region, where so-called “mito-aggresomes” form. However, the mechanisms maintaining these perinuclear clusters remain incompletely understood.

Our data are consistent with a model in which SUMO2/3-associated remodeling may contribute to the organization of perinuclear mitochondrial networks during infection. This interpretation is supported by the time-dependent changes in SUMO2/3 abundance and colocalization with mitochondrial dynamics proteins and MAVS. However, because we did not directly measure SUMOylation of MFN1, DRP1, MAVS, or other mitochondrial targets, we cannot conclude that SUMO2/3 directly retains damaged mitochondria or limits mitophagy. Functional studies will be required to determine whether SUMO2/3 actively regulates mitochondrial quality control, antiviral signaling outputs, or both.

Therefore, the dynamic regulation of SUMO2/3 provides an additional layer connecting mitochondrial remodeling with altered antiviral signaling. The early increase in SUMO2/3 abundance, followed by reduced signal at 96 hpi and altered colocalization with mitochondrial dynamics proteins, suggests that SARS-CoV-2 infection affects post-translational regulatory pathways that may influence both mitochondrial organization and MAVS/RLR signaling outputs. Rather than representing isolated observations, the proteomic, transcriptional, imaging, and cytokine data converge on a model in which SARS-CoV-2 promotes mitochondrial remodeling, ISG15/SUMO-associated post-translational regulation, and a selective inflammatory response with limited interferon production.

## 5. Conclusions

Collectively, our data indicate that SARS-CoV-2 promotes coordinated remodeling of mitochondrial architecture, antiviral transcriptional programs, and post-translational regulatory pathways in lung-derived LC-HK2 cells. These alterations include early mitochondrial stress and fission-associated signatures, followed by late mitochondrial elongation/perinuclear redistribution, increased ISG15 abundance, spatial association between ISG15 and MFN2-positive structures, and dynamic modulation of SUMO2/3-associated signaling.

Importantly, the robust induction of antiviral and ISG15-related transcripts occurred in parallel with limited IFN-α2 secretion and a selective pro-inflammatory cytokine profile, suggesting a partial uncoupling between antiviral transcriptional regulation and downstream interferon output. Thus, the integrated proteomic, transcriptional, cytokine, and imaging data support the involvement of a mitochondrial–immune regulatory axis during SARS-CoV-2 infection, while further functional studies will be required to define the causal mechanisms linking ISG15/SUMO regulation to mitochondrial remodeling and antiviral signaling. In this context, these observations provide a basis for future mechanistic studies investigating how mitochondrial remodeling, ISG15-associated responses, and SUMO-dependent regulation contribute to cellular responses during SARS-CoV-2 infection.

## Figures and Tables

**Table 1:** Summary of the expression profile, biological function, and inferred cellular outcomes of mitochondria-related genes modulated in LC-HK2 cells following SARS-CoV-2 infection.

Gene	Category	Expression Profile	Biological Function	Possible Cellular Outcome	References
*ISG15*	Innate immunity/*ISG*	↑ 48 h and 96 h; perinuclear colocalization with *MFN2* at 96 h	Ubiquitin-like protein; ISGylation	Up: antiviral effector; modulates mitochondrial fusion. Functional inactivation of *MFN2*	[27]
*SUMO2/3*	Post-translational modification	↑ 48 h, ↔ 96 h	SUMOylation of stress and antiviral proteins	Up: early: stress response	[73,74]
*SUMO1*	Post-translational modification	↓ 48 h, ↑ 96 h	SUMOylation regulator of *IFN* signaling and mitochondrial quality control	Down: weakens *IFN* signaling and mitophagy	[25,75]
*MAVS*	Innate immunity adaptor	↑ 48 h and 96 h	Adaptor for *RIG-I/MDA5* antiviral signaling	Up: activation of *IFN-I/III* pathways	[76,77]
*IFIH1* (*MDA5*)	Viral RNA sensor	↑ 48 h and 96 h	Sensor of viral dsRNA	Up: triggers *MAVS-IRF3/7*	[78]
*RIG-I* (*DDX58*)	Viral RNA sensor	↑ 48 h and 96 h	Sensor of viral RNA	Up: triggers *MAVS-IRF3/7*	[78]
*IRF7*	Transcription factor	↑ 48 h and 96 h	Induction of type I *IFN* genes	Up: type I *IFN* response	[79]
*IFIT1*	*ISG*	↑ 48 h and 96 h	Restriction of viral translation	Up: antiviral activity	[79]
*STAT1*	*IFN* signaling	↑ 48 h and 96 h	Mediates *ISG* transcription	Up: denotes a persistence of the antiviral transcriptional program.	[80]
*STAT3*	Transcription factor	↔	Cell survival and signaling	Neutrality of *STAT3* points to a failure in the cellular protection system	[81]
Caspases	Cell death	↑ 48 h and 96 h	Apoptosis effectors	Up: apoptosis induction (may be antagonized by virus)	[82]
*MFN1*	Mitochondrial dynamics	↑ 48 h and 96 h	Mitochondrial fusion and metabolic homeostasis	Up: enhances mitochondrial fusion and OXPHOS capacity	[20,83]
*MFN2*	Mitochondrial dynamics	↓ 48 h, ↑ 96 h	Fusion, ER–mitochondria tethering, *MAVS* modulation	Down: fragmentation; Up: fusion/biogenesis, ER-mitochondria contacts	[20,84]
*DNM1L* (*DRP1*)	Mitochondrial dynamics	↑ 48 h	GTPase driving mitochondrial fission	Up: fragmentation, stress response, apoptosis priming	[24]
*CS*	Mitochondrial metabolism	↑ 96 h	Krebs cycle enzyme; biogenesis marker	Up: increased mitochondrial mass and metabolic flux	[85]
*MT-CO1*	OXPHOS	↑ 48 h and 96 h	Complex IV subunit	Up: compensation for OXPHOS stress	[86]
*MT-CYB*	OXPHOS	↑ 48 h and 96 h	Complex III subunit	Up: supports electron transport	[86]
*MT-ND1*	OXPHOS	↑ 48 h, ↔ 96 h	Complex I subunit	Up: compensatory electron flux; basal later	[14]
*MT-ND5*	OXPHOS	↑ 48 h, ↔ 96 h	Complex I subunit	Up: compensatory electron flux; basal later	[14]
*MT-ND6*	OXPHOS	↑ 48 h, ↔ 96 h	Complex I subunit	Up: compensatory electron flux; basal later	[14]

This table summarizes the expression profile, biological function, and inferred cellular outcomes of mitochondria-related genes modulated in LC-HK2 cells following SARS-CoV-2 infection. Genes involved in mitochondrial fusion (*MFN1* and *MFN2*), fission (*DNM1L*/*DRP1*), metabolic regulation, and biogenesis (*CS*), as well as oxidative phosphorylation (*MT-CO1*, *MT-CYB*, *MT-ND1*, *MT-ND5*, and *MT-ND6*), displayed dynamic time-dependent modulation at 48 and 96 h post-infection (hpi). These coordinated changes suggest an early mitochondrial stress response characterized by fragmentation and compensatory activation of respiratory chain components, followed by partial recovery of mitochondrial fusion, bioenergetic capacity, and biogenesis at later stages of infection. Arrows indicate the expression profile of each gene relative to mock-infected LC-HK2 cells: ↑, increased expression; ↓, decreased expression; and ↔, unchanged expression/no detectable alteration. hpi, hours post-infection. References supporting the functional roles of each gene are indicated by references.

## Data Availability

All mass spectrometry proteomics data have been deposited to Mass Spectrometry Interactive Virtual Environment (MassIVE) Identifier: MSV000093734; password lchk2tcm24. During the review process, the data can be accessed using the following credentials: FTP address: ftp://MSV000093734@massive.ucsd.edu (accessed on 11 April 2026); username: MSV000093734; password: lchk2tcm24.

## Supplementary Materials

The following supporting information can be downloaded at: https://www.mdpi.com/article/10.3390/v18060675/s1. Table S1. Overview of proteomics studies assessing host responses to SARS-CoV-2 infection in lung-derived cell models. Table S2. Primer sequences used for RT-qPCR gene expression analysis in LC-HK2 cells. Table S3. Primary and fluorophore-conjugated secondary antibodies used for immunofluorescence in LC-HK2 cells. Table S4. Proteins identified in Mock-treated samples after 48 hpi. Table S5. Detected differentially abundant proteins of LC-HK2 cells at 48 hpi with SARS-CoV-2 compared to mock-infected cells. Table S6. Detected differentially abundant proteins of LC-HK2 cells at 96 hpi with SARS-CoV-2 compared to mock-infected cells. Table S7. Proteins consistently modulated across independent SARS-CoV-2 proteomics studies. Figure S1. CellProfiler-based segmentation workflow for nuclear, cytoplasmic, perinuclear, and puncta-level analyses of ISG15 and mitofusin signals. Figure S2. Qualitative protein groups uniquely detected in mock- or SARS-CoV-2–infected LC-HK2 cells at 48 hpi.

## Abbreviations

The following abbreviations are used in this manuscript:

ACE2	Angiotensin-converting enzyme 2
ARPC5	Actin-related protein 2/3 complex subunit 5
ATP5IF1	ATP synthase inhibitory factor subunit 1
CCN1	Cellular communication network factor 1
CETN2	Centrin 2
CHCHD2	Coiled-coil-helix-coiled-coil-helix domain containing 2
COVID-19	Coronavirus disease 2019
DIABLO	Mitochondrial diablo homolog
DRP1	Dynamin-related protein 1
FDR	False discovery rate
FIS1	Mitochondrial fission protein 1
GO	Gene Ontology
GPX1	Glutathione peroxidase 1
hpi	Hours post-infection
IFI35	Interferon-induced protein 35
IFIH1	Interferon induced with helicase C domain 1
IFIT1	Interferon-induced protein with tetratricopeptide repeats 1
IFIT3	Interferon-induced protein with tetratricopeptide repeats 3
IL	Interleukin
IRF7	Interferon regulatory factor 7
ISG15	Interferon-stimulated gene 15
LC-HK2	Human non-small cell lung carcinoma cells
LLPS	Liquid–liquid phase separation
MAVS	Mitochondrial antiviral-signaling protein
MDA-5	Melanoma differentiation-associated protein 5
MT-ND1	Mitochondrially encoded NADH/ubiquinone oxidoreductase core subunit 1
MTCH2	Mitochondrial carrier homolog 2
NDUFS5	NADH dehydrogenase [ubiquinone] iron–sulfur protein 5
NFKB2	Nuclear factor kappa B subunit 2
NFX1	NFX1-type zinc finger-containing protein 1
ORF	Open reading frame
OXPHOS	Oxidative phosphorylation
P4HA2	Prolyl 4-hydroxylase subunit alpha 2
PLpro	Papain-like protease
pp1ab	Replicase polyprotein 1ab
PTMs	Post-translational modifications
RAB1B	RAS-associated protein RAB1B
RIG-I	Retinoic acid-inducible gene I
RPL37A	Ribosomal protein L37a
RT-qPCR	Reverse transcription quantitative polymerase chain reaction
SARS-CoV-2	Severe acute respiratory syndrome coronavirus 2
SMAD3	SMAD family member 3
STAT1	Signal transducer and activator of transcription 1
SUMO	Small ubiquitin-like modifier
SUMO1	Small ubiquitin-like modifier 1
SUMO2/3	Small ubiquitin-like modifier 2/3
TBK1	TANK-binding kinase 1
TMPRSS2	Transmembrane serine protease 2
TNFɑ	Tumor necrosis factor alpha
TRIM28	Tripartite motif containing 28
ΔΨm	Mitochondrial membrane potential
