# Supplementary material for: Exploring Key Regulators of Mitochondrial Dynamics and Immune Response in SARS-CoV-2 Infection
Source: Viruses. 2026 Jun 16;18(6):675. doi: 10.3390/v18060675 (PMC13307764; doi:10.3390/v18060675)
Supplement: Supplementary file 1 [file viruses-18-00675-s001.zip › Table S1.pdf]

Supplementary 1; Table S1. Overview of proteomics studies assessing host responses to SARS-CoV-2 infection in lung-derived cell models

Study	Cell line	Strain	MOI	Time post-infection	MS quantification
**Babačić, 2023** **[58]**	Human lung adenocarcinoma Calu-3	SARS-CoV-2 ancestral variant	1	1 and 3 days	TMT
**Stukalov, 2021** **[60]**	A549 cells	SARS-CoV-Frankfurt-1 or SARS-CoV-2-MUC-IMB-1 strains	2	6, 12, 24 hours	DIA
**Hatton, 2021** **[62]**	Adult primary human nasal airway epithelial cells	SARS-CoV-2 (BetaCoV/England/2/2020)	0.1	72 hours	TMT
**Puray-Chavez, 2021** **[59]**	ACE2-negative H522 human lung cells	SARS-CoV-2 strain 2019-nCoV/USA-WA1/2020	1	4, 12, 24, 48, 72, 96 hours	TMT
**Grossegesse, 2022** **[61]**	Calu-3	SARS-CoV (strain Hong Kong) or SARS-CoV-2 (hCoV-19/Italy/INMI1-isl/2020)	5	2, 6, 10, 24 hours	DIA

**Crozier, 2022**<br><br>**[57]**	hAEC-ALI ciliated cells and Calu-3	SARS-CoV-2/human/Liverpool/REMRQ0001/2020 or SARS-CoV-2 England/ATACCC 174/2020	<0.1 for Calu-3 cells<br><br>MOI >3 for hAEC-ALI ciliated cells	72 hours for hARC-ALI<br><br>8, 24, 48 hours for Calu-3	TMT

Supplementary 1; Table S1. This table summarizes key experimental parameters from published mass spectrometry–based proteomics studies investigating host cellular responses to SARS-CoV-2 infection in lung-derived cell systems. For each study, the lung-related cell type, viral strain, multiplicity of infection (MOI), post-infection sampling time points, and proteomic quantification strategy are reported. Time points are presented as hours or days post-infection, as stated in the original publications. TMT, tandem mass tag labeling; DIA, data-independent acquisition.
