# Supplementary material for: Exploring Key Regulators of Mitochondrial Dynamics and Immune Response in SARS-CoV-2 Infection
Source: Viruses. 2026 Jun 16;18(6):675. doi: 10.3390/v18060675 (PMC13307764; doi:10.3390/v18060675)
Supplement: Supplementary file 1 [file viruses-18-00675-s001.zip › Table S2.pdf]

Supplementary 2; Table S2: Primer sequences used for expression analysis by qRT-PCR in LC- HK2

Target gene	Functional category	Gene ID	Primer	Sequence (5'–3')
ACE2	Viral entry receptor	59272	Forward	AAACATACTGTGACCCCGCAT
ACE2	Viral entry receptor	59272	Reverse	CCAAGCCTCAGCATATTGAACA
TMPRSS2	Viral entry receptor	7113	Forward	AATCGGTGTGTTCGCCTCTAC
TMPRSS2	Viral entry receptor	7113	Reverse	CGTAGTTCTCGTTCCAGTCGT
RIG-I (DDX58)	Innate immunity (RLR)	23586	Forward	CTTTTTCTCAAGTTCCTGTTGGA
RIG-I (DDX58)	Innate immunity (RLR)	23586	Reverse	TCCCAACTTTCAATGGCTTC
MDA5 (IFIH1)	Innate immunity (RLR)	64135	Forward	GGCACCATGGGAAGTGATT
MDA5 (IFIH1)	Innate immunity (RLR)	64135	Reverse	GATGATGATATTCTTCCCTTCCA
STAT1	IFN signaling	6772	Forward	TCGGGGAATATTCAGAGCAC
STAT1	IFN signaling	6772	Reverse	CCAGGCTCTTGATTTCATGC
ISG15	IFN-stimulated gene	9636	Forward	CTCTGAGCATCCTGGTGAGGAA
ISG15	IFN-stimulated gene	6772	Reverse	AAGGTCAGCCAGAACAGGTCGT
SUMO1	SUMOylation	7341	Forward	TCAACTGAGGACTTGGGGGA
SUMO1	SUMOylation	7341	Reverse	TCAGCAATTCTCTGACCCTCA
SUMO2	SUMOylation	6613	Forward	AGGATGGTTCTGTGGTGCAG
SUMO2	SUMOylation	6613	Reverse	CCATTTCCAACTGTCGTTCACA
MFN1	Mitochondrial fusion	55669	Forward	ACTTCCTTCTGCAGCTGTGT
MFN1	Mitochondrial fusion	55669	Reverse	AGCTGGCTGTCTTGTACGTG
MFN2	Mitochondrial fusion	9927	Forward	TGACATCTGTGCCTGGACTG
MFN2	Mitochondrial fusion	9927	Reverse	TACCGAGGGCTCAGAGGAAA
DNM1L (DRP1)	Mitochondrial fission	10059	Forward	TCGCTGTCACTGCTGCTAAT
DNM1L (DRP1)	Mitochondrial fission	10059	Reverse	GCATCAGTACCCGCATCCAT
MT-ND1	OXPHOS / Complex I	4535	Forward	TCATGACCCTTGGCCATAAT
MT-ND1	OXPHOS / Complex I	4535	Reverse	CTAGTTCGGACTCCCCTTCG
MT-ND5	OXPHOS / Complex I	4540	Forward	ACTGTTCATCGGCTGAGAGG
MT-ND5	OXPHOS / Complex I	4540	Reverse	GCTAAGGCGAGGATGAAACC
MT-ND6	OXPHOS / Complex I	4541	Forward	TTCTGAATTTTGGGGGAGGT
MT-ND6	OXPHOS / Complex I	4541	Reverse	CCCCATGCCTCAGGATACTC
MT-CYB	OXPHOS / Complex III	4519	Forward	CCACCCCATCCAACATCTCC
MT-CYB	OXPHOS / Complex III	4519	Reverse	GCGTCTGGTGAGTAGTGCAT
MT-COI	OXPHOS / Complex IV	4512	Forward	CCAATACCAAACGCCCCTCT
MT-COI	OXPHOS / Complex IV	4512	Reverse	TGTTGAGGTTGCGGTCTGTT
CS	Mitochondrial biogenesis	1431	Forward	CCCTTTCCGACCCTTACCTG
CS	Mitochondrial biogenesis	1431	Reverse	ACTTCCTTCTGCAGCTGTGT
SDHA	Mitochondrial biogenesis	6389	Forward	TGCCATCCACTACATGACGG
SDHA	Mitochondrial biogenesis	6389	Reverse	GCTCTGTCCACCAAATGCAC
RPL37A	Endogenous control	6168	Forward	ATTGAAATCAGCCAGCACGC
RPL37A	Endogenous control	6168	Reverse	AGGAACCACAGTGCCAGATCC
