# Supplementary material for: Exploring Key Regulators of Mitochondrial Dynamics and Immune Response in SARS-CoV-2 Infection
Source: Viruses. 2026 Jun 16;18(6):675. doi: 10.3390/v18060675 (PMC13307764; doi:10.3390/v18060675)
Supplement: Supplementary file 1 [file viruses-18-00675-s001.zip › Table S3.pdf]

Supplementary 3; Table S3. Primary and secondary antibodies used for immunofluorescence analyses in LC-HK2 cells

Antibody (target / specificity)	Host / Type	Dilution	Supplier
**Primary Antibodies**			
Recombinant anti-ISG15 (EPR3446)	Rabbit monoclonal	1:100	Abcam, MA, USA
Anti-RIG-I (ab111037)	Goat polyclonal	1:200	Abcam, MA, USA
Anti-MDA5 (ab4544)	Goat polyclonal	1:200	Abcam, MA, USA
Anti-mitofusin 2 (MFN2; ab56889-6A8)	Mouse monoclonal	1:200	Abcam, MA, USA
Anti-ACE2 (ab655863)	Rabbit polyclonal	1:200	Abcam, MA, USA
Anti-SARS-CoV-2 N protein (ab272852)	Mouse monoclonal	1:500	Abcam, MA, USA
Anti-SARS-CoV-2 S protein (ab272504)	Rabbit monoclonal	1:500	Abcam, MA, USA
Anti-MAVS (3993)	Rabbit polyclonal	1:300	Cell Signaling Technology, MA, USA
**Secondary antibodies**			
FITC-conjugated donkey anti-rabbit IgG (H+L) (sc-2090)	Donkey	1:1000	Santa Cruz Biotechnology, TX, USA
CFL 647–conjugated donkey anti-rabbit IgG (H+L) (sc-362291)	Donkey	1:1000	Santa Cruz Biotechnology, TX, USA
Alexa Fluor™ 647–conjugated goat anti-rabbit IgG (A21245)	Goat	1:1000	Life Technologies, NY, USA
Alexa Fluor™ Plus 488–conjugated goat anti-mouse IgG (A-11001)	Goat	1:1000	Life Technologies, NY, USA
Texas Red–conjugated donkey anti-goat IgG (H+L) (ab6883)	Donkey	1:1000	Abcam, MA, USA
Alexa Fluor™ 488–conjugated donkey anti-goat IgG (H+L) (A-11055)	Donkey	1:1000	Thermo Fisher Scientific, MA, USA
FITC-conjugated goat anti-human IgG (F0132)	Goat	1:1000	Sigma-Aldrich, MO, USA

Legend: List of primary and fluorophore-conjugated secondary antibodies used for immunofluorescence staining in LC-HK2 cells. Primary antibodies target viral, mitochondrial, and innate immune proteins, while secondary antibodies were selected according to the host species of each primary antibody. n.d., not determined.
