# Supplementary material for: Exploring Key Regulators of Mitochondrial Dynamics and Immune Response in SARS-CoV-2 Infection
Source: Viruses. 2026 Jun 16;18(6):675. doi: 10.3390/v18060675 (PMC13307764; doi:10.3390/v18060675)
Supplement: Supplementary file 1 [file viruses-18-00675-s001.zip › viruses-4285142-Supplementary Materials-update_revised.pdf]

Supplementary Materials

Table S1. Overview of proteomics studies assessing host responses to SARS-CoV-2 infection in lung-derived cell models.

Study	Cell line	Strain	MOI	Time post-infection	MS quantification
Babačić, 2023 [57]	Human lung adenocarcinoma Calu-3	SARS-CoV-2 ancestral variant	1	1 and 3 days	TMT
Stukalov, 2021 [59]	A549 cells	SARS-CoV-Frankfurt-1 or SARS-CoV-2-MUC-IMB-1 strains	2	6, 12, 24 hours	DIA
Hatton, 2021 [61]	Adult primary human nasal airway epithelial cells	SARS-CoV-2 (BetaCoV/England/2/2020)	0.1	72 hours	TMT
Puray-Chavez, 2021 [58]	ACE2-negative H522 human lung cells	SARS-CoV-2 strain 2019-nCoV/USA-WA1/2020	1	4, 12, 24, 48, 72, 96 hours	TMT
Grossegesse, 2022 [60]	Calu-3	SARS-CoV (strain Hong Kong) or SARS-CoV-2 (hCoV-19/Italy/INMI1-isl/2020)	5	2, 6, 10, 24 hours	DIA
Crozier, 2022 [56]	hAEC-ALI ciliated cells and Calu-3	SARS-CoV-2/human/Liverpool/REMRQ0001/2020 or SARS-CoV-2 England/ATACCC 174/2020	<0.1 for Calu-3 cells<br><br>MOI >3 for hAEC-ALI ciliated cells	72 hours for hARC-ALI<br><br>8, 24, 48 hours for Calu-3	TMT

This table summarizes key experimental parameters from published mass spectrometry–based proteomics studies investigating host cellular responses to SARS-CoV-2 infection in lung-derived cell systems. For each study, the lung-related cell type, viral strain, multiplicity of infection (MOI), post-infection sampling time points, and proteomic quantification strategy are reported. Time points are presented as hours or days post-infection, as stated in the original publications. TMT, tandem mass tag labeling; DIA, data-independent acquisition.

Table S2. Primer sequences used for RT-qPCR gene expression analysis in LC-HK2 cells.

Target gene	Functional category	Gene ID	Primer	Sequence (5'–3')
ACE2	Viral entry receptor	59272	Forward	AAACATACTGTGACCCCGCAT
ACE2	Viral entry receptor	59272	Reverse	CCAAGCCTCAGCATATTGAACA
TMPRSS2	Viral entry receptor	7113	Forward	AATCGGTGTGTTCGCCTCTAC
TMPRSS2	Viral entry receptor	7113	Reverse	CGTAGTTCTCGTTCCAGTCGT

RIG-I (DDX58)	Innate immunity (RLR)	23586	Forward	CTTTTTCTCAAGTTCCTGTTGGA
RIG-I (DDX58)	Innate immunity (RLR)	23586	Reverse	TCCCAACTTTCAATGGCTTC
MDA5 (IFIH1)	Innate immunity (RLR)	64135	Forward	GGCACCATGGGAAGTGATT
MDA5 (IFIH1)	Innate immunity (RLR)	64135	Reverse	GATGATGATATTCTTCCCTTCCA
STAT1	IFN signaling	6772	Forward	TCGGGGAATATTCAGAGCAC
STAT1	IFN signaling	6772	Reverse	CCAGGCTCTTGATTTCATGC
ISG15	IFN-stimulated gene	9636	Forward	CTCTGAGCATCCTGGTGAGGAA
ISG15	IFN-stimulated gene	6772	Reverse	AAGGTCAGCCAGAACAGGTCGT
SUMO1	SUMOylation	7341	Forward	TCAACTGAGGACTTGGGGGA
SUMO1	SUMOylation	7341	Reverse	TCAGCAATTCTCTGACCCTCA
SUMO2	SUMOylation	6613	Forward	AGGATGGTTCTGTGGTGCAG
SUMO2	SUMOylation	6613	Reverse	CCATTTCCAACTGTCGTTCACA
MFN1	Mitochondrial fusion	55669	Forward	ACTTCCTTCTGCAGCTGTGT
MFN1	Mitochondrial fusion	55669	Reverse	AGCTGGCTGTCTTGTACGTG
MFN2	Mitochondrial fusion	9927	Forward	TGACATCTGTGCCTGGACTG
MFN2	Mitochondrial fusion	9927	Reverse	TACCGAGGGCTCAGAGGAAA
DNM1L (DRP1)	Mitochondrial fission	10059	Forward	TCGCTGTCACTGCTGCTAAT
DNM1L (DRP1)	Mitochondrial fission	10059	Reverse	GCATCAGTACCCGCATCCAT
MT-ND1	OXPHOS / Complex I	4535	Forward	TCATGACCCTTGGCCATAAT
MT-ND1	OXPHOS / Complex I	4535	Reverse	CTAGTTCGGACTCCCCTTCG
MT-ND5	OXPHOS / Complex I	4540	Forward	ACTGTTCATCGGCTGAGAGG
MT-ND5	OXPHOS / Complex I	4540	Reverse	GCTAAGGCGAGGATGAAACC
MT-ND6	OXPHOS / Complex I	4541	Forward	TTCTGAATTTTGGGGGAGGT
MT-ND6	OXPHOS / Complex I	4541	Reverse	CCCCATGCCTCAGGATACTC
MT-CYB	OXPHOS / Complex III	4519	Forward	CCACCCCATCCAACATCTCC
MT-CYB	OXPHOS / Complex III	4519	Reverse	GCGTCTGGTGAGTAGTGCAT
MT-COI	OXPHOS / Complex IV	4512	Forward	CCAATACCAAACGCCCCTCT
MT-COI	OXPHOS / Complex IV	4512	Reverse	TGTTGAGGTTGCGGTCTGTT
CS	Mitochondrial biogenesis	1431	Forward	CCCTTTCCGACCCTTACCTG
CS	Mitochondrial biogenesis	1431	Reverse	ACTTCCTTCTGCAGCTGTGT
SDHA	Mitochondrial biogenesis	6389	Forward	TGCCATCCACTACATGACGG
SDHA	Mitochondrial biogenesis	6389	Reverse	GCTCTGTCCACCAAATGCAC
RPL37A	Endogenous control	6168	Forward	ATTGAAATCAGCCAGCACGC
RPL37A	Endogenous control	6168	Reverse	AGGAACCACAGTGCCAGATCC

This table lists the target genes analyzed (grouped by functional category), corresponding gene IDs, and the forward and reverse primer sequences (5′ –3′) used for RT-qPCR assays. The panel includes genes associated with viral entry (ACE2, TMPRSS2), innate immune sensing and IFN signaling (RIG-I/DDX58, MDA5/IFIH1, STAT1, ISG15), SUMOylation (SUMO1, SUMO2), and mitochondria dynamics/respiratory function (MFN1, MFN2, DNM1L/DRP1, MT-ND1, MT-ND5, MT-ND6, MT-CYB, MT-COI, CS, SDHA), as well as the endogenous control RPL37A.

Table S3. Primary and fluorophore-conjugated secondary antibodies used for immunofluorescence in LC-HK2 cells.

Antibody (target / specificity)	Host / Type	Dilution	Supplier
**Primary Antibodies**			
Recombinant anti-ISG15 (EPR3446)	Rabbit monoclonal	1:100	Abcam, MA, USA
Anti-RIG-I (ab111037)	Goat polyclonal	1:200	Abcam, MA, USA
Anti-MDA5 (ab4544)	Goat polyclonal	1:200	Abcam, MA, USA
Anti-mitofusin 2 (MFN2; ab56889-6A8)	Mouse monoclonal	1:200	Abcam, MA, USA
Anti-ACE2 (ab655863)	Rabbit polyclonal	1:200	Abcam, MA, USA
Anti-SARS-CoV-2 N protein (ab272852)	Mouse monoclonal	1:500	Abcam, MA, USA
Anti-SARS-CoV-2 S protein (ab272504)	Rabbit monoclonal	1:500	Abcam, MA, USA
Anti-MAVS (3993)	Rabbit polyclonal	1:300	Cell Signaling Technology, MA, USA
**Secondary antibodies**			
FITC-conjugated donkey anti-rabbit IgG (H+L) (sc-2090)	Donkey	1:1000	Santa Cruz Biotechnology, TX, USA
CFL 647–conjugated donkey anti-rabbit IgG (H+L) (sc-362291)	Donkey	1:1000	Santa Cruz Biotechnology, TX, USA
Alexa Fluor™ 647–conjugated goat anti-rabbit IgG (A21245)	Goat	1:1000	Life Technologies, NY, USA
Alexa Fluor™ Plus 488–conjugated goat anti-mouse IgG (A-11001)	Goat	1:1000	Life Technologies, NY, USA
Texas Red–conjugated donkey anti-goat IgG (H+L) (ab6883)	Donkey	1:1000	Abcam, MA, USA
Alexa Fluor™ 488–conjugated donkey anti-goat IgG (H+L) (A-11055)	Donkey	1:1000	Thermo Fisher Scientific, MA, USA
FITC-conjugated goat anti-human IgG (F0132)	Goat	1:1000	Sigma-Aldrich, MO, USA

This table lists the antibodies used for immunofluorescence staining of LC-HK2 cells, including target/specificity, host species and clonality, working dilution, and supplier. Primary antibodies were

selected to detect viral antigens (SARS-CoV-2 S and N), mitochondri-al/mitochondria-associated proteins (MFN2, MAVS), and innate immune sensors/effectors (ISG15, RIG-I, MDA5, ACE2). Fluorophore-conjugated secondary antibodies (FITC, Alexa Fluor™ 488, Alexa Fluor™ 647, Texas Red, and CFL 647) were chosen according to the host species of each primary antibody to enable multi-channel confocal imaging. n.d., not determined.

Table S4. Proteins identified in Mock-treated samples after 48 hpi. Samples were submitted to LC-MSMS analysis, and the protein identification was carried out in PEAKS Studio X software.

Only proteins identified in at least two biological replicates are depicted. Proteins identified in SARS-COV-2-infected samples after 48hpi. (Mock 48hpi). Samples were submitted to LC-MSMS analysis and the protein identification was carried out in PEAKS software. Only proteins identified in at least two biological replicates are depicted. (SARS-CoV-2 48hpi). Proteins identified in Mock-treated samples 96 hpi. Samples were submitted to LC-MSMS analysis and the protein identification was carried out in PEAKS software. Only proteins identified in at least two biological replicates are depicted (Mock 96hpi). Proteins identified in SARS-COV-2 infected samples after 96hpi. Samples were submitted to LC-MSMS analysis and the protein identification was carried out in PEAKS software. Only proteins identified in at least two biological replicates are depicted (SARS-CoV-2 48hpi).

Table S5. Detected differentially abundant proteins of LC-HK2 cells at 48 hpi with SARS-CoV-2 compared to mock-infected cells.

Detected differentially abundant proteins of LC-HK2 cells at 96 hpi with SARS-CoV2 compared to Mock-infected cells. Label free quantification of proteins was performed using Maxquant Platform. Data was processed in Perseus, proteins that were only identified by site, reverse sequences and contaminants were filtered out. Only proteins identified in at least 3 acquisitions of each group were considered for comparison, remaining missing values were imputed from normal distribution. The statistical analysis was performed using Limma R-package. Proteins that showed p-value < 0.05 and $0.585 >$ log2 fold change < 0.585 were considered to be differentially abundant.

Table S6. Detected differentially abundant proteins of LC-HK2 cells at 96 hpi with SARS-CoV-2 compared to mock-infected cells.

Detected differentially abundant proteins of LC-HK2 cells at 48 hpi with SARS-CoV2 compared to Mock-infected cells. Label free quantification of proteins was performed using Maxquant Platform. Data was processed in Perseus, proteins that were only identified by site, reverse sequences and contaminants were filtered out. Only proteins identified in at least 3 acquisitions of each group were considered for comparison, remaining missing values were imputed from normal distribution. The statistical analysis was performed using Limma R-package. Proteins that showed p-value < 0.05 and $0.585 >$ log2 fold change < 0.585 were considered to be differentially abundant.

Table S7. Proteins consistently modulated across independent SARS-CoV-2 proteomics studies.

This table provides the complete list of proteins identified as differentially modulated in at least four independent proteomics studies comparing SARS-CoV-2–infected vs mock lung-derived cell models. For each protein, the table reports the corresponding identifiers and the studies in which it was

detected as differentially regulated, enabling identification of robust, cross-study signals associated with SARS-CoV-2 infection.

Figure S1. CellProfiler-based segmentation workflow for nuclear, cytoplasmic, perinuclear, and puncta-level analyses of ISG15 and mitofusin signals.

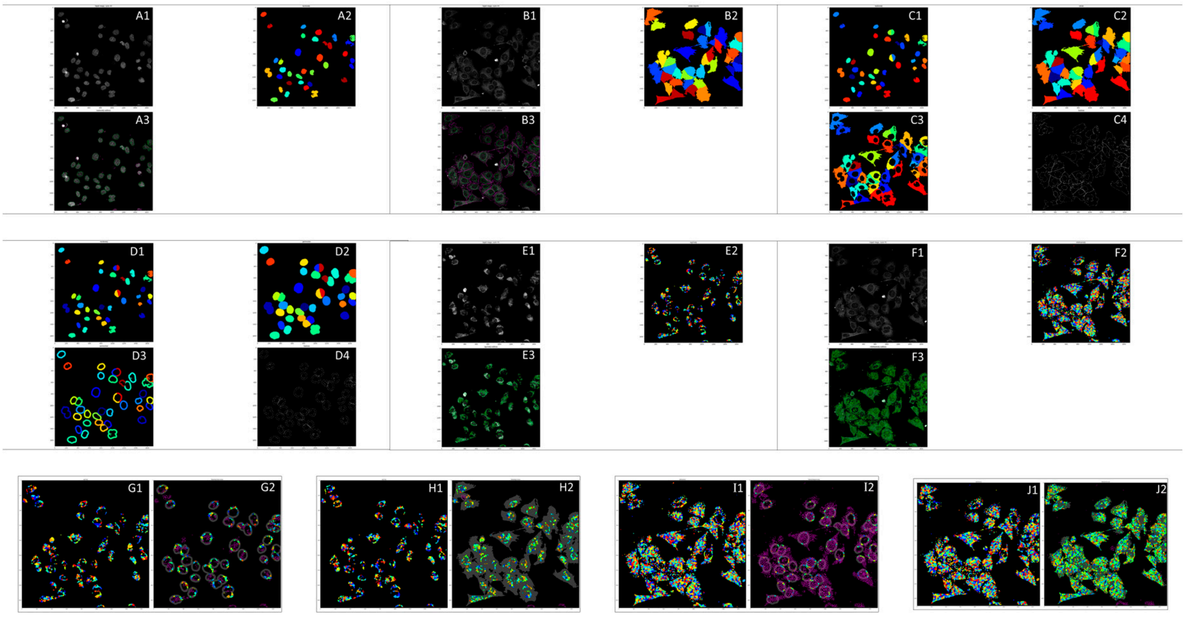

Figure S1. CellProfiler-based segmentation workflow for nuclear, cytoplasmic, perinuclear, and puncta-level analyses of ISG15 and mitofusin signals. Fluorescence images were processed in CellProfiler™ 4.2 to define cellular compartments and to segment ISG15- and mitofusin-positive puncta used in downstream quantification. (A1–A3) Nuclei identification from Hoechst staining: raw nuclear channel (A1), automated nuclear masks (A2), and segmentation outlines (A3). (B1–B3) Whole-cell segmentation guided by ISG15 signal dispersion using segmented nuclei as seeds: mitofusin channel example (B1), whole-cell masks (B2), and overlay of nuclear (green) and whole-cell (magenta) out-lines (B3). (C1–C4) Cytoplasm definition by subtracting nuclear masks from whole-cell masks: nu-clear masks (C1), whole-cell masks (C2), cytoplasmic masks (C3), and cytoplasmic outlines (C4). (D1–D3) Perinuclear compartment generation: nuclear mask (D1), expanded nucleus + sur-rounding region (D2), and perinuclear ring obtained by subtracting the nuclear mask (D3). (E1–E3) ISG15 puncta segmentation: raw ISG15 channel (E1), segmented ISG15-positive aggregates (E2), and outlines (E3). (F1–F3) Mitofusin puncta segmentation: raw mitofusin channel (F1), segmented mitofusin-positive aggregates (F2), and outlines (F3). (G–J) Spatial assignment of puncta to cellular compartments: ISG15 puncta overlaid on the perinuclear region (G1–G2) or across the entire cell area (H1–H2), and mitofusin puncta localized within the perinuclear region (I1–I2) or distributed across the whole-cell area (J1–J2).

Figure S2. Qualitative protein groups uniquely detected in mock- or SARS-CoV-2–infected LC-HK2 cells at 48 hpi.

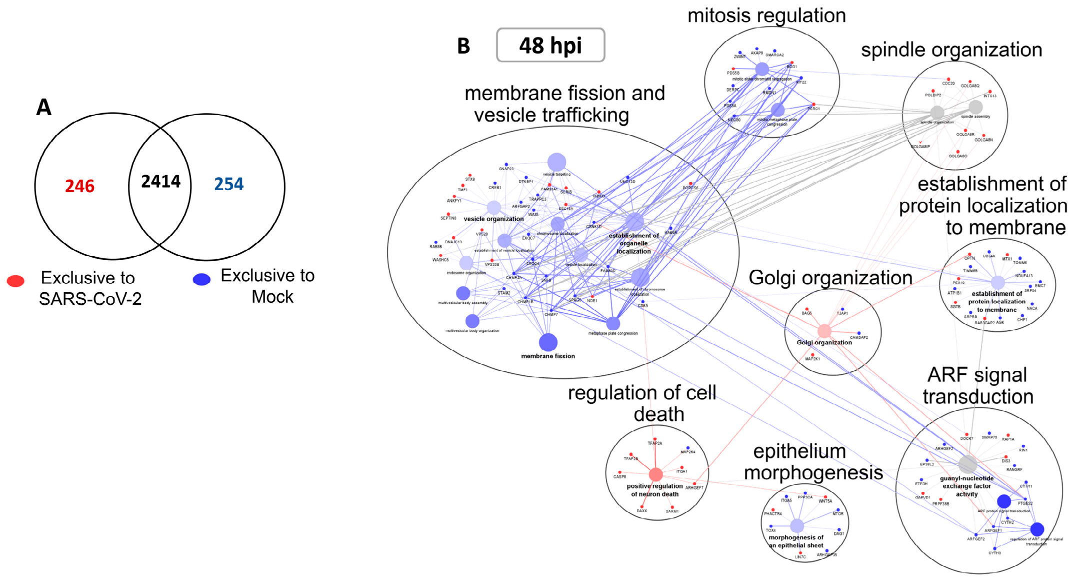

Figure S2. Qualitative protein groups uniquely detected in mock- or SARS-CoV-2–infected LC-HK2 cells at 48 hpi. (A) Venn diagram summarizing protein groups detected exclusively in SARS-CoV-2 (red; n = 246) or mock (blue; n = 254) conditions at 48 h post-infection (hpi); n = 2414 protein groups were shared between conditions. (B) Network representation of enriched biological processes/pathways derived from the exclusive protein sets at 48 hpi. Nodes represent enriched terms (node size pro-portional to significance after multiple-testing correction), and small labeled points indicate repre-sentative proteins contributing to each term; edges denote shared proteins between terms. Color coding indicates enrichment driven by proteins exclusive to SARS-CoV-2 (red) or mock (blue). Major functional modules include membrane fission and vesicle trafficking, Golgi organization, mitosis regulation/spindle organization, establishment of protein localization to membrane, ARF signal transduction, regulation of cell death, and epithelium morphogenesis.
