# Supplementary figures and images for: Exploring Key Regulators of Mitochondrial Dynamics and Immune Response in SARS-CoV-2 Infection
Source: Viruses. 2026 Jun 16;18(6):675. doi: 10.3390/v18060675 (PMC13307764; doi:10.3390/v18060675)

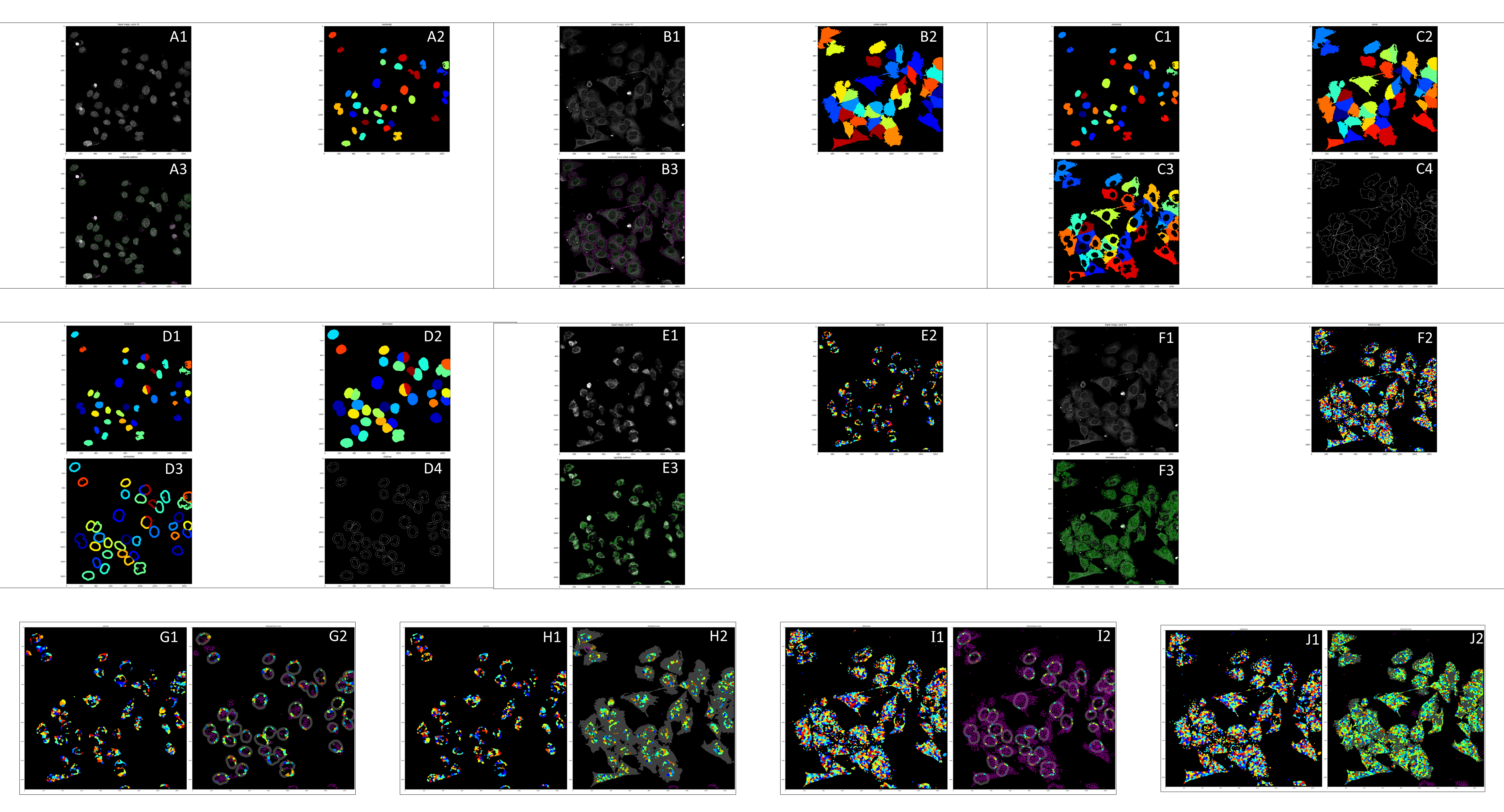

Supplement: Supplementary file 1 [file viruses-18-00675-s001.zip › Figure S1.tif]
